# ERF49 mediates brassinosteroid regulation of heat stress tolerance in *Arabidopsis thaliana*

**DOI:** 10.1186/s12915-022-01455-4

**Published:** 2022-11-10

**Authors:** Xia Chen, Huidan Xue, Liping Zhu, Huiqin Wang, Hao Long, Jun Zhao, Funing Meng, Yunfei Liu, Yuan Ye, Xiaomin Luo, Zhi Liu, Guanghui Xiao, Shengwei Zhu

**Affiliations:** 1 Key Laboratory of Plant Molecular Physiology, Institute of Botany, Chinese Academy of Sciences, Beijing, 100093 China; 2 College of Life Science, University of Chinese Academy of Sciences, Beijing, 100049 China; 3 School of Food and Biological Engineering, Shaanxi University of Science and Technology, Xi’an, 710021 China; 4 College of Life Sciences, Shaanxi Normal University, Xi’an, 710119 China; 5 College of Bioscience and Biotechnology, Hunan Agricultural University, Changsha, 410128 China

**Keywords:** ERF49, Heat stress, Brassinosteroid (BR), Brassinazole resistant 1 (BZR1), *Arabidopsis thaliana*

## Abstract

**Background:**

Heat stress is a major abiotic stress affecting the growth and development of plants, including crop species. Plants have evolved various adaptive strategies to help them survive heat stress, including maintaining membrane stability, encoding heat shock proteins (HSPs) and ROS-scavenging enzymes, and inducing molecular chaperone signaling. Brassinosteroids (BRs) are phytohormones that regulate various aspects of plant development, which have been implicated also in plant responses to heat stress, and resistance to heat in *Arabidopsis thaliana* is enhanced by adding exogenous BR. Brassinazole resistant 1 (BZR1), a transcription factor and positive regulator of BR signal, controls plant growth and development by directly regulating downstream target genes. However, the molecular mechanism at the basis of BR-mediated heat stress response is poorly understood. Here, we report the identification of a new factor critical for BR-regulated heat stress tolerance.

**Results:**

We identified ERF49 in a genetic screen for proteins required for BR-regulated gene expression. We found that *ERF49* is the direct target gene of BZR1 and that overexpressing *ERF49* enhanced sensitivity of transgenic plants to heat stress. The transcription levels of heat shock factor *HSFA2*, heat stress-inducible gene *DREB2A*, and three heat shock protein (HSP) were significantly reduced under heat stress in *ERF49-*overexpressed transgenic plants. Transcriptional activity analysis in protoplast revealed that BZR1 inhibits *ERF49* expression by binding to the promoter of *ERF49*. Our genetic analysis showed that dominant gain-of-function *brassinazole resistant 1-1D* mutant (*bzr1-1D*) exhibited lower sensitivity to heat stress compared with wild-type. Expressing *ERF49-SRDX* (a dominant repressor reporter of ERF49) in *bzr1-1D* significantly decreased the sensitivity of *ERF49-SRDX/bzr1-1D* transgenic plants to heat stress compared to *bzr1-1D*.

**Conclusions:**

Our data provide clear evidence that BR increases thermotolerance of plants by repressing the expression of *ERF49* through BZR1, and this process is dependent on the expression of downstream heat stress-inducible genes. Taken together, our work reveals a novel molecular mechanism mediating plant response to high temperature stress.

**Supplementary Information:**

The online version contains supplementary material available at 10.1186/s12915-022-01455-4.

## Background

As sessile and autotrophic organisms, terrestrial plants are constantly challenged by unsuitable environmental changes. Plants respond and adapt to environmental changes primarily by regulating external morphology and internal physiological characteristics. High temperature stress during plant growth and development processes caused by global warming is a critical issue in agriculture and ecology. Heat stress leads to severely reduced crop yield and degraded quality [1]. In plants, high temperatures induce changes in morphology, anatomy, and biochemistry at all levels of organization. At very high temperatures (more than 40 °C for *Arabidopsis*), severe cellular injury can occur and rapidly cause cell death. At moderately high temperatures (around 28 to 37 °C), obvious deleterious effects in plant growth and reproduction occur over a relatively long time [2]. Injuries caused by high temperature are mainly due to the denatured and aggregated proteins, increased membrane lipid fluidity, inactivated enzymes, altered microtubule organization, and disrupted cytoskeleton structures [3, 4]. Heat stress may reduce ion flux and increase production of reactive oxygen species (ROS) and other toxic compounds, which severely affect plant growth [3, 5].

To cope with heat stress, plants have evolved various mechanisms, including maintaining membrane stability, encoding heat shock proteins (HSPs) and ROS-scavenging enzymes, and inducing molecular chaperone signaling [6]. In higher plants, HSPs are extremely rapidly and intensively induced in a wide variety of cells and organisms to trigger thermotolerance. Five classes of HSPs have been distinguished by molecular weight: HSP100, HSP90, HSP70, HSP60, and small HSPs (15–30 kDa) [7, 8]. Many studies assert that the contribution of HSPs to heat tolerance is to act as molecular chaperones to maintain and/or restore the homeostasis of cell proteins under heat stress. For instance, HSP70 and HSP90 facilitate a variety of important processes including protein folding, protein transport, oligomeric protein assembly, and reactivation of denatured proteins [9, 10]. Moreover, heat shock factors (HSFs) are the key components mediating the activation of genes responsive to heat stress. They recognize and bind heat shock elements (HSEs), which are palindromic binding motifs located in the promoters of heat shock genes, conferring plant thermotolerance [11]. The 21 HSFs encoded in the *A. thaliana* genome can be categorized into three major classes (HSFA, HSFB, HSFC), based on the peculiarities of their oligomerization domain [12].

Brassinosteroids (BRs), polyhydroxylated steroidal phytohormones, regulate diverse aspects of plant biology, including elongation and division of cells, root development, stomatal and vascular differentiation, and seed germination [13, 14]. BRs also mediate plant responses to stress, including drought, salinity, disease, heat, cold, and nutrient deficiency [15, 16]. Numerous components of BR signaling have been identified in plants [17, 18]. BZR1 (Brassinazole resistant 1) is a positive regulator of BR signaling with dual roles in activating BR responses and regulating negative feedback of BR biosynthesis [19]. BZR1 activates BR responses by modulating its direct target genes [20, 21]. According to a previous study, control of thermomorphogenesis by phytochrome interacting factor 4 (PIF4) is dependent on BZR1 activity [22]. They found that BZR1 is a true, temperature-dependent positive regulator of *PIF4*, acting as a major growth coordinator. However, the molecular mechanisms of BZR1 and BR signaling pathways that mediate plant response to high temperature stress are still unclear.

The ERF (ethylene responsive factors) family is one of the largest families of transcription factors involved in a variety of biological processes related to growth and development, as well as various responses to environmental stimuli [23]. ERFs, belonging to the AP2/ERF multi-gene family, are known to be the integrative node in ethylene signaling and in the regulation of several stress-responsive genes and also common transcription factors of different signaling pathways in *Arabidopsis* [24]. Ectopic expression of a number of *ERF* genes enhances the tolerance of plants to heat stress, indicating that ERFs participate in heat stress response [25, 26]. However, the role of ERF-mediated heat stress response remains unclear. In this study, we identified ERF49 in a genetic screen for proteins critical to BR-regulated gene expression. ERF49 is one member of AP2/ERF family which functions directly in diverse plant developmental and physiological processes. We showed that *ERF49* promoter physically interacts with BZR1, which inhibits the expression of *ERF49*. Dominant inhibition of ERF49 enhanced the thermotolerance of plants by increasing the expression of downstream heat stress-inducible genes under high temperature, and ERF49 participated in the plant response to high temperature mainly through the BZR1 pathway. Together, our results identified an important transcription factor in process of how BR-enhanced plants cope with heat stress, laying a foundation for in-depth studies on the molecular mechanism of BR-enhanced thermotolerance in plants and further molecular improvement of crop tolerance to high temperature conditions.

## Results

### BR enhanced heat stress tolerance in Arabidopsis thaliana

**Fig. 1:**
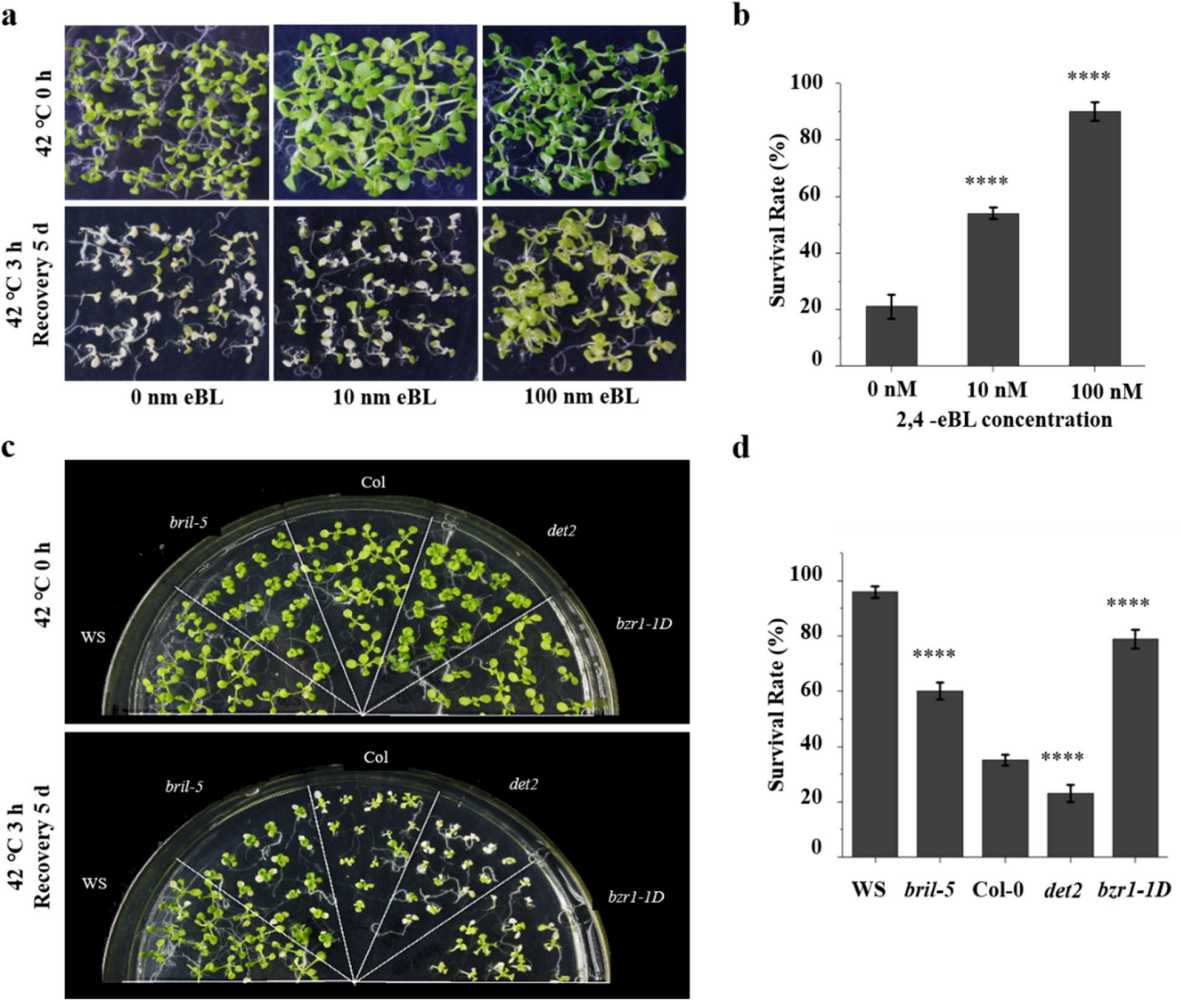
Exogenous 2, 4-eBL treatment and endogenous BR increase thermotolerance of *Arabidopsis thaliana.* **a** The influence of different concentrations of 2, 4-eBL on heat tolerance of *Arabidopsis thaliana*. **b** Survival rate of *A. thaliana* treated with 2, 4-eBL in a heat stress environment. **c** The thermotolerance of BR biosynthesis and signaling transduction mutants. **d** The survival rate of BR biosynthesis and signaling transduction mutants after heat stress treatment. Seven-day-old seedlings were treated under 42 °C for 3 h and recovery for 5 days. Sixty to eighty seedlings of each genotype from three biological replicates were counted. Asterisks represent statistical significance (****, *P* < 0.0001; one-way ANOVA followed by post hoc Tukey’s multiple comparison test). Numerical data is provided in Additional file 6. Experiments were repeated for three times, the error bars indicate standard deviation

One study has shown that applying exogenous 2, 4- epi-brassinosteroid (eBL) increases the heat resistance of rape and tomato seedlings [27] and reported that BR increased the basal heat resistance of *A. thaliana* seedlings [28]. To verify these results, we first examined the effect of exogenous application of 2, 4-eBL on the response of *A. thaliana* Col-0 to high temperature. Compared with the control group, the survival rate of *A. thaliana* treated with 10 nM 2, 4-eBL increased significantly under high temperature stress (54.4 %), while the survival rate of *A. thaliana* treated with 100 nM 2, 4-eBL was the highest (92.2 %) (Fig. 1a, b).

To further study the effect of endogenous BR on plant response to high temperature, we examined the response of BR-related mutants in *A. thaliana* to high temperature. As shown in Fig. 1c and d, *det2* (in the *Arabidopsis* ecotype Columbia (Col-0) background) and *bri1-5* (in the *Arabidopsis* ecotype Wassilewskija (WS) background) plants were more sensitive to high temperature compared with wild-type plants. *det2* and *bri1-5* had lower survival rates under high temperature stress (respectively 23 %, 60 %) than wild-type plants (35 %, 96 %), while the dominant gain-of-function mutant *bzr1-1D* was less sensitive to high temperature and had higher survival rates (79 %) than wild-type Col-0 (35 %) under high temperature stress. Together, the results indicate that BR enhances the tolerance of plants to high temperature and that overexpression of *BZR1* can enhance BR-induced heat stress tolerance.

### ERF49 structure prediction and characterization

Based on the chromatin immunoprecipitation microarray (ChIP-chip) analysis of BZR1, it was found that almost 1/3 of the members of AP2/ERF family are BR-regulated BZR1 target (BRBT) genes [21]. Many studies showed that the AP2/ERF transcription factor family is closely related to plant response to stress [29–31]. Further expression analysis of *BZR1* and target genes in *AP2/ERF* family by *Arabidopsis* eFP Browser showed that the absolute expression of *ERF49* and *BZR1* is consistently similar in most tissues (Additional file 1: Fig. S1), revealing that *ERF49* may be the target gene of BZR1 and play the role in plant resistance to stress.

**Fig. 2:**
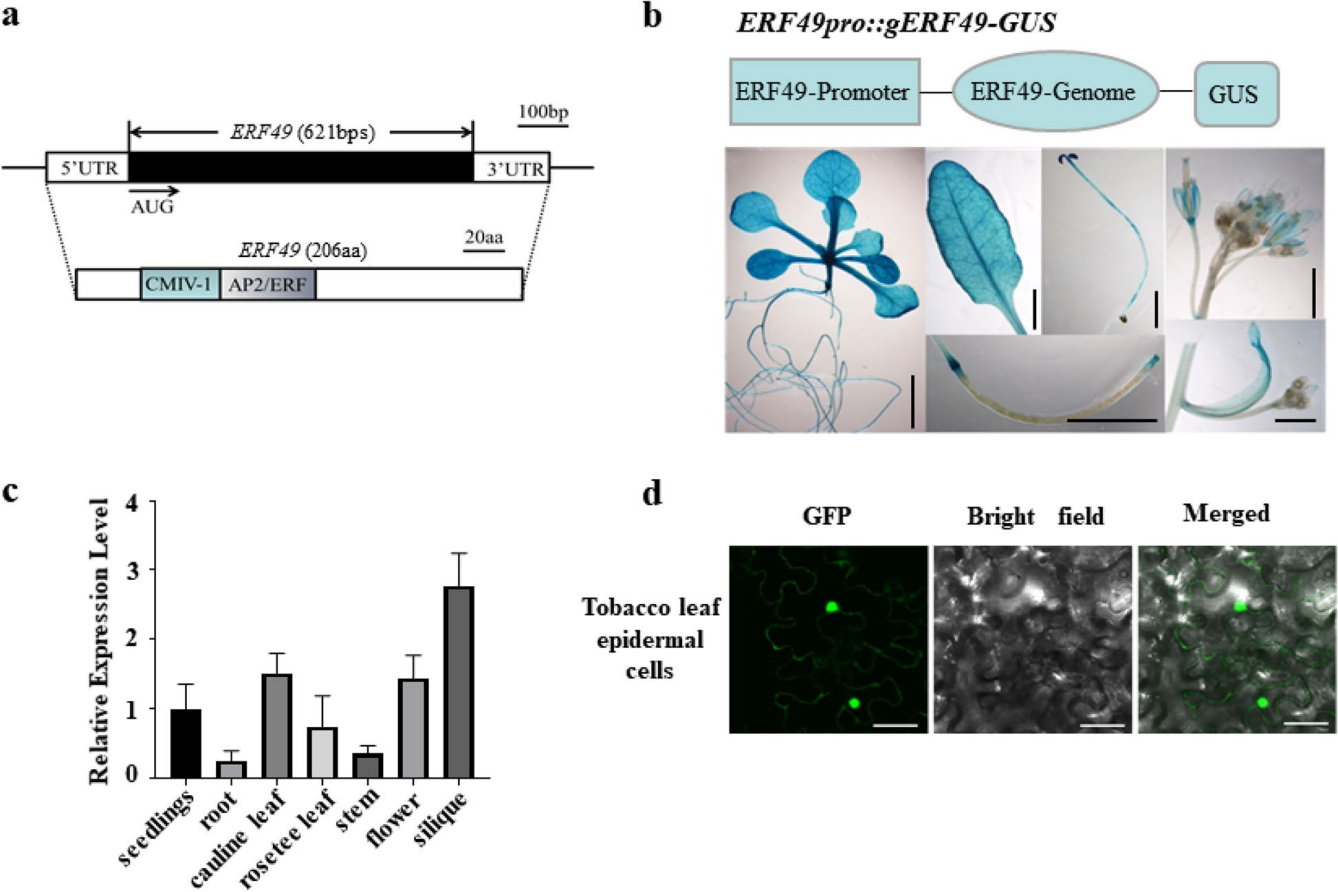
The structure and characterization of ERF49. **a** The gene and protein structure of ERF49. The exon is denoted by a black box. 5′-UTR and 3′-UTR are denoted by white boxes. The translation initiation codons are shown. aa means amino acids and bp means base pair. **b** GUS staining assay of *ERF49pro::gERF49-GUS* transgenic plants in different tissues (from left to right: 14-day-old seedling, rosette leaf (upper), 5-day-old seedling (upper), silique (lower), inflorescence (upper), and cauline leaf (lower)). Scale bars, 5 mm. **c** Analysis of *ERF49* expression level in different organs by quantitative reverse transcription-polymerase chain reaction (RT-qPCR). The expression level in the seedlings is set to 1.0, and error bars represent standard deviations of three biological replicates. Numerical data is provided in Additional file 6. **d** Confocal micrographs of subcellular localization of reporter signal exhibited by *35S::ERF49-GFP* in tobacco leaf. Scale bars: 50 μm

ERF49 protein belongs to group IV according to analysis of the phylogeny and conserved domains of the AP2/ERF family in *Arabidopsis* and is one member of the DREB subfamily of the AP2/ERF family [23]. Members of DREB subfamily specifically bind to the dehydration-responsive element (DRE)/C-repeat (CRT) *cis*-acting element (A-GCCGAC) and control the gene expression responsive to drought and low-temperature stress in *Arabidopsis* [32]. ERF49 is 206 amino acids long and has a molecular weight of 22.6 kDa. As predicted, it contains a single DNA-binding domain (the AP2/ERF domain) and the motif CMIV-1 (Fig. 2a). CMIV-1 is 27 amino acid residues defined by the conserved amino acid motif [K/R] GKGGPxN and predicted to contain a nuclear localization sequence (NLS) [33].

To further investigate the characterization of ERF49 in *A. thaliana*, we generated transgenic *Arabidopsis* seedlings expressing a *ERF49pro::gERF49-GUS* transgene. The results of GUS staining showed that ERF49 protein was mainly accumulated in seedlings, hypocotyl, and rosette leaves and weakly accumulated in stems, inflorescences, silique, and sepals (Fig. 2b). The expression analysis using quantitative reverse transcription PCR (RT-qPCR) showed that *ERF49* was highly expressed in seedlings, siliques, leaf, and flower (Fig. 2c). Furthermore, ERF49-GFP (green fluorescent protein) fusion protein transiently expressed from the constitutive 35S promoter (*35S::ERF49-GFP*) in tobacco leaf cells showed that fluorescent signals were mainly in nuclei (Fig. 2d).

### ERF49 is a negative regulator in plant thermotolerance

**Fig. 3:**
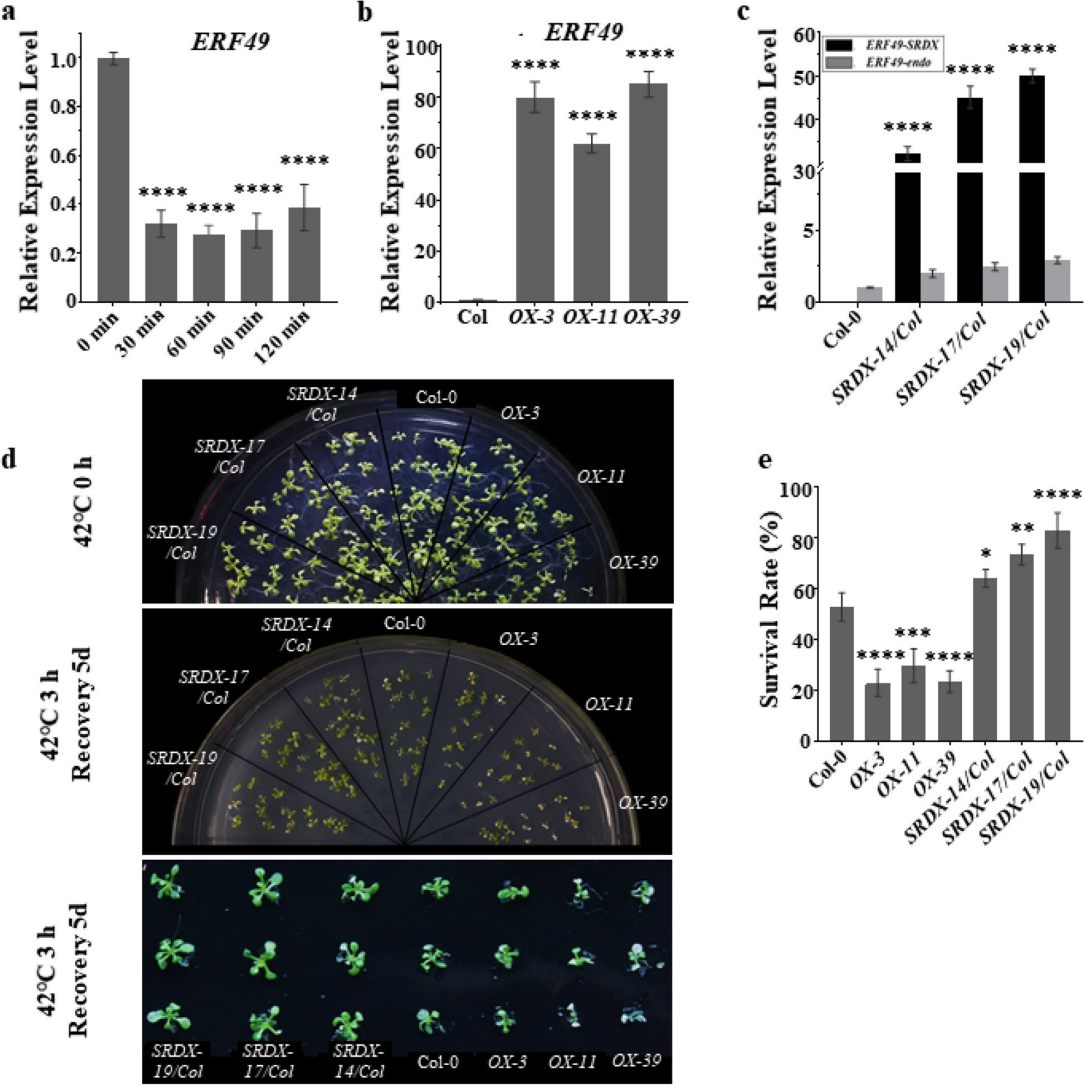
ERF49 affects heat stress responses in *Arabidopsis thaliana.* ***a*** The relative expression level of *ERF49* in *Arabidopsis* seedlings after heat treatment for 0, 30, 60, 90, and 120 min in a 42°C environment. **b** The relative expression level of *ERF49* in three *ERF49*-overexpression transgenic lines. **c** The relative expression level of *ERF49* in three *ERF49*-dominant-negative transgenic lines. **d** The phenotype of *ERF49*-overexpression and dominant-negative transgenic lines after heat stress treatment at 42°C for 0 h (top panel) and 3 h and recovery for 5 days (middle and bottom panels). The bottom panel shows the phenotype of 3 random seedlings in each *ERF49* transgenic line in the middle panel graph. Col-0 is control. **e** The survival rates of *ERF49* overexpression and dominant-negative transgenic lines after heat stress treatment at 42°C for 3 h and recovery for 5 days; 60–80 seedlings of each genotype from three biological replicates were counted. Col-0 is control. Asterisks represent statistical significance (*, *P* < 0.05; **, *P* < 0.01; ***, *P* < 0.001; ****, *P* < 0.0001; one-way ANOVA followed by post hoc Tukey’s multiple comparison test). *UBC30* was used as a reference gene. Numerical data is provided in Additional file 6. The experiments were repeated three times, error bars indicate standard deviation

Our RT-qPCR analysis revealed that the expression of *ERF49* was decreased at the transcriptional level after high temperature treatment (Fig. 3a), indicating that ERF49 might be a negative regulator in heat stress responses. In order to test this speculation, we generated transgenic plants overexpressing *ERF49* (*ERF49-OX*) or *ERF49* fused to the SRDX transcriptional repression domain driven by the *CaMV*35S promoter (*ERF49-SRDX*) in the Col-0 background. The SRDX motif is the modified version of the EAR-motif repression domain to generate a chimeric repressor from the transcription factors. This dominant repressor also represses the activity of the functionally redundant homologs [34, 35]. The relative expression levels of *ERF49* transgenic plants under Col-0 are shown in Fig. 3b and c. The relative expression of *ERF49* in overexpressing lines OX*-3*, OX*-11*, and OX*-39* was significantly increased compared with Col-0 (Fig. 3b). *ERF49-SRDX* expression was also significantly increased in *SRDX-14*, *SRDX-17*, and *SRDX-19*, indicating that it significantly suppressed the activity of ERF49 (Fig. 3c). Analysis of the survival rate showed that the overexpressed transgenic plants decreased in size and survival rate (mean about 25 %), compared with wild-type (mean about 52 %) after high temperature treatment. The dominant inhibited transgenic plants were larger in overall size than the wild-type plants, and their survival rate (mean about 73 %) was significantly higher than that of the wild-type plants (Fig. 3d, e).

Considering that the induction of HSPs is a central component of responses to heat stress in plants, we determined the expression levels of several representative *HSPs* in transgenic plants (Additional file 1: Fig. S2a-c). The expression of *HSP26.5*, *HSP70*, and *HSP90.1* were slightly different in transgenic plants under control conditions. Differently, the transcript levels of these *HSPs* in *ERF49-OX* plants were significantly reduced compared with the wild-type under heat stress. In contrast, transcript levels of these *HSPs* were significantly higher in *ERF49-SRDX* plants than in wild-type plants under heat stress. Since heat induces *HSPs* mainly via HSFs, we also examined whether ERF49 regulates *HSFs* expression. The results showed that the expression of *HSFA2* and heat stress-inducible gene *DREB2A* in *ERF49-OX* plants were significantly reduced, but were significantly induced in *ERF49-SRDX* plants under heat stress (Additional file 1: Fig. S2d, e). Taken together, we speculated that ERF49 negatively regulates the tolerance of plants to high temperature by reducing the expression of downstream heat stress-inducible genes.

### ERF49 is a direct target gene of BZR1, and its expression is repressed by BR and BZR1

**Fig. 4:**
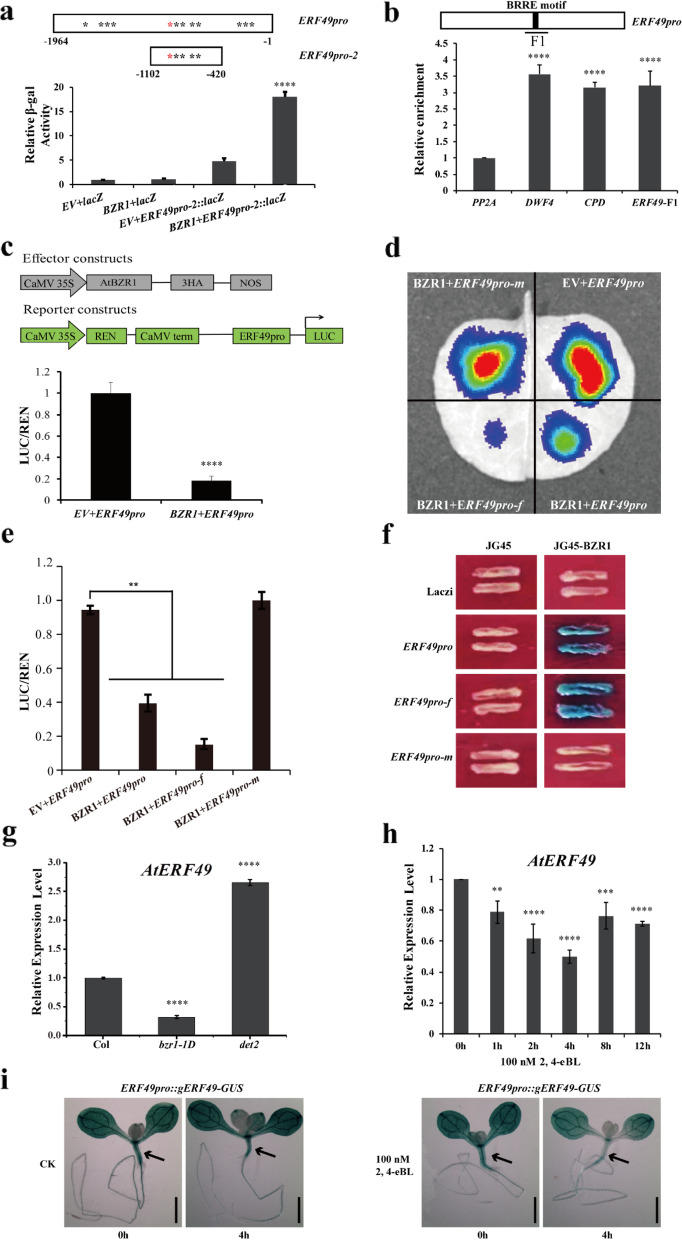
Expression of *ERF49*, a direct target gene of BZR1, is repressed by BZR1 and BR. **a** Yeast one-hybrid assays for binding of BZR1 to the *ERF49* promoter. Diagram depicting the segmentation of *ERF49* promoter. Black and red asterisks indicate the positions of putative E-box and BRRE motifs, respectively. The region from −1964 to −1 bp was named *ERF49pro* and the region from −1102 to −420 bp was named *ERF49pro-2* (upper panel). The activity of β-galactosidase when BZR1 and *ERF49pro-2* were transformed into the yeast cells together (lower panel). **b** ChIP-qPCR assays for direct binding of BZR1 to the *ERF49* promoter. Relative enrichment of *PP2A* coding region was normalized as an internal control. *DWF4* and *CPD* were identified as target genes of BZR1. ERF49-F1 indicates the sequence analyzed by ChIP-qPCR. **c** Dual-luciferase reporter assay of *ERF49* in *Arabidopsis* protoplasts. Schematic diagram of effector (*35S::BZR1*) and reporter (*ERF49pro::LUC*) constructs used in the protoplast transcription system (upper panel). Expression activity assay of LUC reporter genes in *Arabidopsis* protoplasts transformed with the effector and reporter plasmids (lower panel). The plasmid *pUC18-3HA* was empty vector (EV). **d** Transcriptional activity assays of the interaction between BZR1 protein and *ERF49* promoter fragments. Tobacco transient expression of the LUC reporter gene (under the control of the *ERF49* promoter fragments with native or mutant BRRE motif) by the BZR1. **e** Quantification of the dual-luciferase assays of LUC expression in **d**. The expression of REN (Renilla luciferase) was used as the internal control. The LUC/REN ratio indicates the relative activity of the promoter. Asterisks represent statistical significance (**, *P* < 0.01; one-way ANOVA followed by post hoc Tukey’s multiple comparison test). **f** Yeast one-hybrid assay of the interaction between BZR1 protein and *ERF49* promoter fragments. Systematic yeast one-hybrid assay showing the binding of BZR1 to the *ERF49* promoter fragments with native or mutant BRRE motif. **g** The relative expression level of *ERF49* in Col-0, *bzr1-1D*, and *det2.* **h** Relative expression level of *ERF49* in Col-0 after 100 nM 2, 4-eBL for 0, 1, 2, 4, 8, and 12 h. **i** GUS activities of the *ERF49pro::gERF49-GUS* transgenic *Arabidopsis* plants after soaking in a solution of 100 nM 2, 4-eBL for 0 and 4 h. CK was a solution containing 80% ethanol. Scale bars, 2 mm. Asterisks represent statistical significance (**, *P* < 0.01; ***, *P* < 0.001; ****, *P* < 0.0001; one-way ANOVA followed by post hoc Tukey’s multiple comparison test). *UBC30* was used as a reference gene. Numerical data is provided in Additional file 6. The experiments were repeated three times, error bars indicate standard deviation

*ERF49* has been identified as BR-regulated BZR1 target gene by transcript profiling and ChIP-chip analysis [21] (Additional file 1: Fig. S3a). We further analyzed the promoter sequence of *ERF49* and found that there are 12 putative BZR1 binding sites in *ERF49* promoter: one BRRE (CGTGT/CG) and 11 E-BOX (CANNTG) [21, 36, 37] (Additional file 1: Fig. S3b). To verify the regulatory effect of BZR1 on *ERF49*, we conducted the yeast one-hybrid assay with BZR1 as prey and *ERF49* promoter which divided into three fragments according to the distribution of BZR1 binding sites as bait. The results of assay showed that lacZ reporter gene activity was strongly activated when BZR1 and the second promoter fragment (*ERF49pro-2*) were simultaneously transformed into the yeast cells (Fig. 4a), indicating that BZR1 binds to the −420 to −1102 bp region of *ERF49* promoter. In ChIP-qPCR assays, the F1 fragment with BRRE motif of *ERF49* promoter sequence was enriched after immunoprecipitation of CFP-tagged BZR1 (Fig. 4b). A transcriptional activity assay demonstrated that co-expression with BZR1 reduced the *LUC* reporter gene activity driven by P2 fragment with BRRE motif. However, the *LUC* reporter gene activity driven by fragment with mutated BRRE motif was comparable to that in control (Fig. 4c–e). Consistently, the yeast one-hybrid experiment also showed that BZR1 interacted with P2 fragment with BRRE motif. The interaction was abolished when BRRE motif in *ERF49* promoter fragment was mutated (Fig. 4f). These results indicate the BZR1 specifically binds to BRRE motif of *ERF49* promoter. RT-qPCR analysis showed that *ERF49* transcription was significantly decreased in the BR signal-enhanced dominant mutant *bzr1-1D*, but significantly increased in the BR-deficient mutant *det2* compared with Col-0 (Fig. 4g). This pattern is consistent with suppression of the transcript level of *ERF49* in Col-0 after treatment with 100 nM 2, 4-eBL (Fig. 4h). We also found that ERF49 protein level decreased in *ERF49pro::gERF49-GUS* transgenic plants after BR treatment by GUS staining (Fig. 4i). Taken together, these results provide direct evidence for BZR1 binding to the promoter of *ERF49* gene in vivo and show that *ERF49* is repressed by BR through BZR1.

### ERF49 is a positive regulator in the BR pathway

**Fig. 5:**
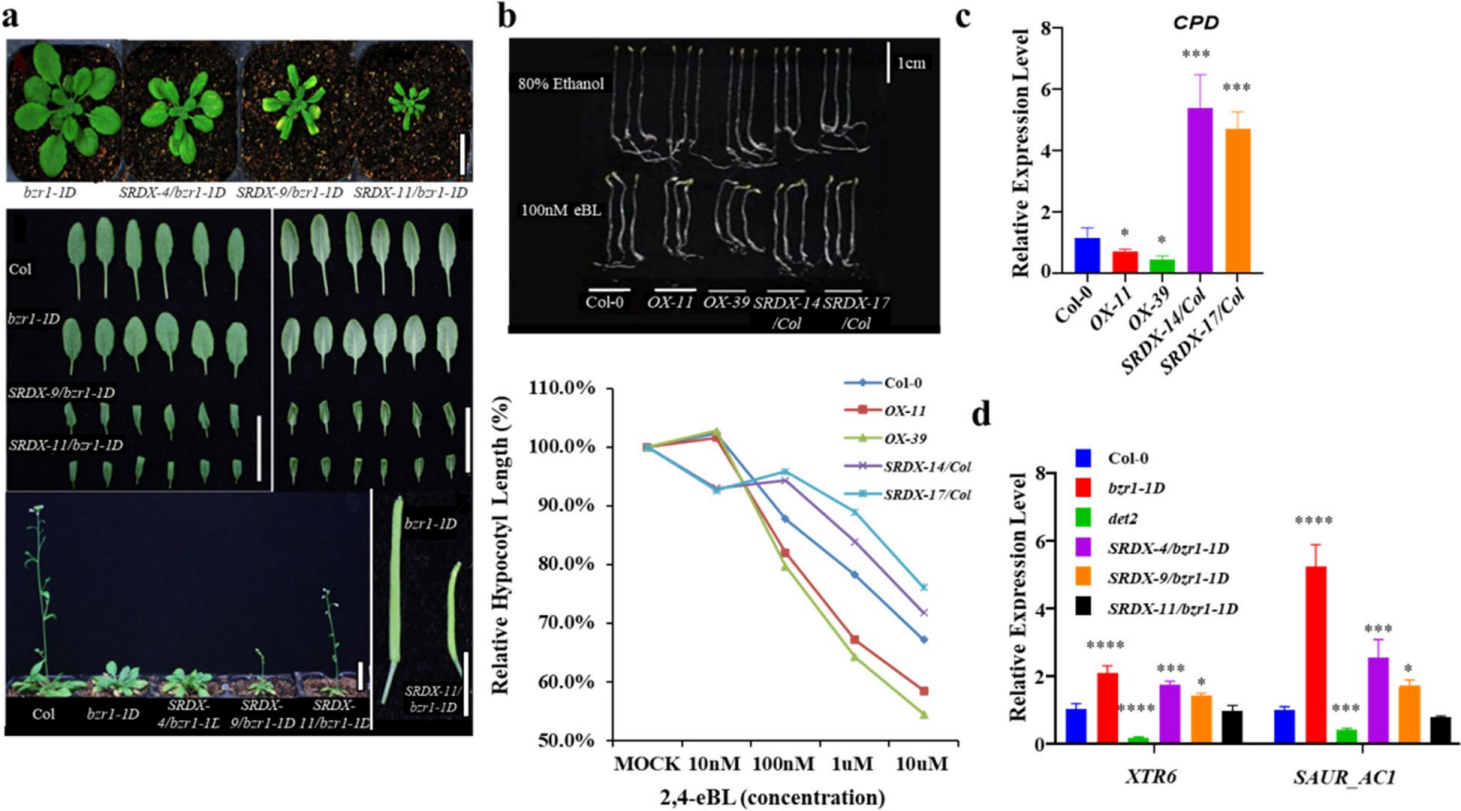
ERF49 is a positive regulator in the BR pathway. **a** The phenotype of *ERF49* dominant-negative transgenic lines in a *bzr1-1D* background. Scale bar for the siliques, 5 mm. The rest of scale bar, 2.5 cm. **b** Analysis of hypocotyl elongation in the *ERF49* transgenic plants with 2, 4-eBL or 80% ethanol (as control) treatment. Scale bar, 1 cm. Hypocotyl lengths of over 30 seedlings were measured using ImageJ for each independent line. The standardized data is shown with mock as 100% (lower panel). **c** Relative expression level of downstream gene *CPD* regulated by BR in the *ERF49* transgenic lines. **d** Relative expression level of BR-regulated downstream genes *XTR6 and SAUR_AC1* in *bzr1-1D*, *det2*, and *ERF49* dominant-negative transgenic lines. Asterisks represent statistical significance (*, *P* < 0.05; ***, *P* < 0.001; ****, *P* < 0.0001; one-way ANOVA followed by post hoc Tukey’s multiple comparison test). *UBC30* was used as a reference gene. Numerical data is provided in Additional file 6. The experiments were repeated three times, error bars indicate standard deviation

In order to further understand the role of ERF49 in BR signal transduction pathway, we generated the transgenic plants driven by *CaMV*35S promoter that fused *ERF49* into the SRDX transcriptional repression domain in the *bzr1-1D* background (*ERF49-SRDX/bzr1-1D*). The relative expression level of transgenic plants is shown in Additional file 1: Fig. S4a. Firstly, we observed the above-ground growth of *ERF49-SRDX/bzr1-1D* transgenic plants. As shown in Fig. 5a, *ERF49-SRDX/bzr1-1D* transgenic lines displayed significantly smaller, narrowed, and curled down leaves with shorter petioles compared to *bzr1-1D*. Moreover, compared with Col-0, the flowering time of *bzr1-1D* mutant was delayed by about 1 week, but overexpression of *ERF49-SRDX* in *bzr1-1D* significantly promoted the flowering time of transgenic plants, close to that of the wild-type Col-0. In addition, distinct from *bzr1-1D*, the transgenic plants also showed shorter and curved siliques (Fig. 5a).

Low-concentration BL promotes hypocotyl elongation, while high-concentration BL inhibits elongation [19, 38, 39]. Analysis of hypocotyl elongation in *ERF49* transgenic plants showed that hypocotyls of *ERF49-OX* plants were shorter than those of Col-0 on medium containing 100 nM 2, 4-eBL (Fig. 5b and Additional file 1: Fig. S4b, c), indicating that overexpression of *ERF49* increased sensitivity to BR. RT-qPCR analysis showed that the expression level of the BR-responsive gene *CPD*, reported to be downregulated by BZR1 [36], decreased in *ERF49-OX* plants compared with Col-0, but increased in *ERF49-SRDX* plants (Fig. 5c). Two downstream response genes of BR signal (*SaurAC1* and *XTR6*), which have very low expression in *det2*, were significantly induced in *bzr1-1D*, and reduced in *ERF49-SRDX/bzr1-1D* transgenic plants but still significantly higher than *det2* (Fig. 5d). Together, these results suggest that ERF49 partially mediates multiple developmental, physiological, and molecular phenotypes and functions as a positive regulator in the BR signaling pathway.

### BZR1 is involved in BR-mediated plant thermotolerance through ERF49 in Arabidopsis thaliana

Since overexpression of *BZR1* enhanced BR-induced plant thermotolerance (Fig. 1c, d) and *ERF49* is the direct target gene of BZR1, we decided to investigate whether the response of ERF49 to heat stress was regulated by BZR1. Here, we examined the response of *ERF49-SRDX/bzr1-1D* transgenic plants to heat stress, the survival analysis showed that the survival rate of *ERF49-SRDX/bzr1-1D* transgenic plants (around 60 %) was significantly higher than *bzr1-1D* (around 40 %) after 3 h heat stress treatment (Additional file 1: Fig. S5).

**Fig. 6:**
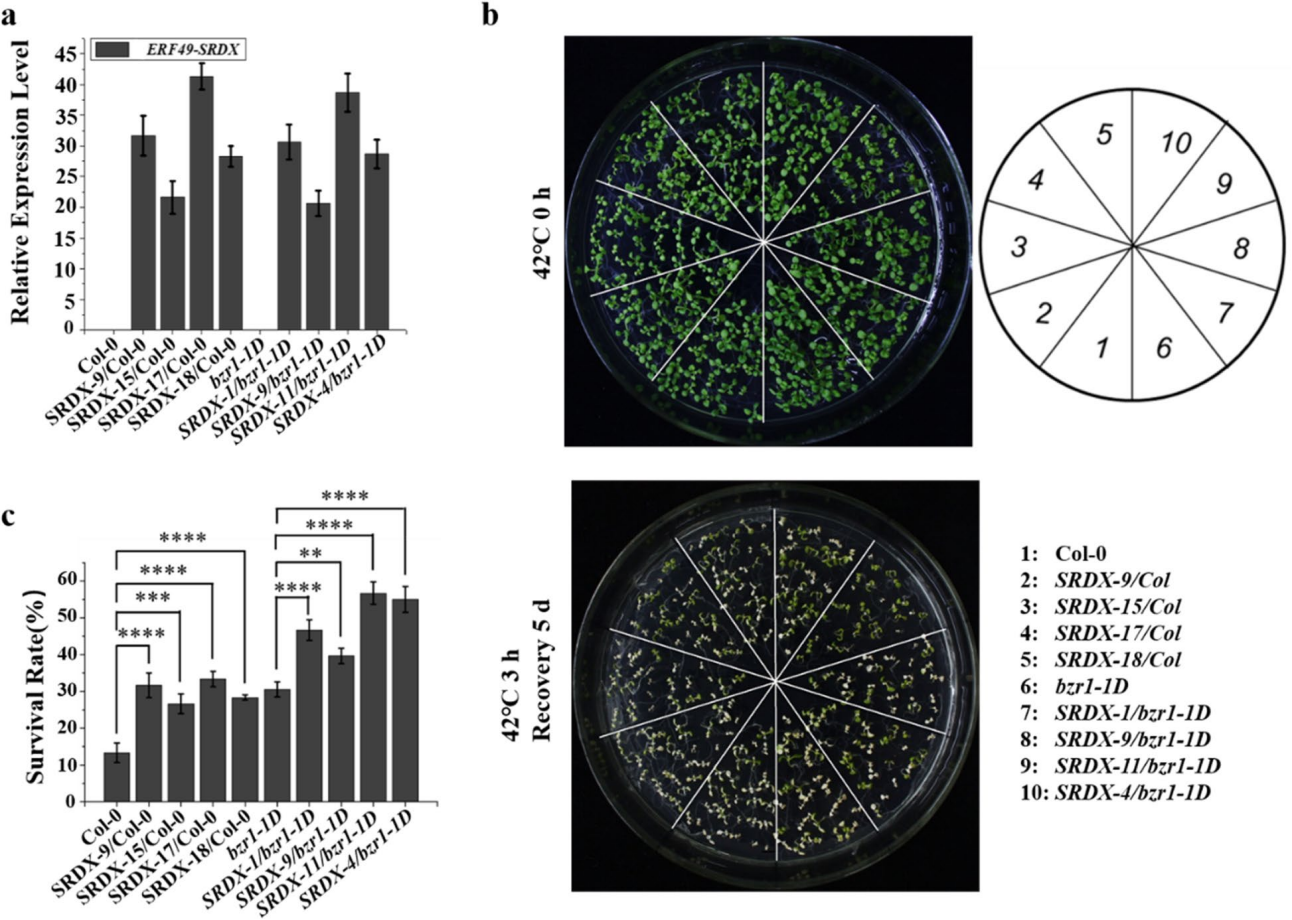
The response of dominant-negative *ERF49* in Col-0 or *bzr1-1D* to heat stress. **a** The relative expression level of *ERF49* in eight *ERF49* dominant-negative transgenic lines. **b, c** The phenotype and survival rates of *ERF49* dominant-negative transgenic lines in Col-0 or *bzr1-1D* after heat stress treatment at 42 °C for 3 hours and recovery for 5 days. Sixty to eighty seedlings of each genotype from three biological replicates were counted. Col-0 and *bzr1-1D* were used as controls. Asterisks represent statistical significance (**, *P*<0.01; ***, *P* < 0.001; ****, *P* < 0.0001; one-way ANOVA followed by post hoc Tukey’s multiple comparison test). Numerical data is provided in Additional file 6. The experiments were repeated three times, error bars indicate standard deviation

Furthermore, we selected four *ERF49*-*SRDX*/Col transgenic lines (*SRDX-9/*Col, *SRDX-15/*Col, *SRDX-17/*Col, *SRDX-18/*Col) and four *ERF49*-*SRDX/bzr1-1D* transgenic lines (*SRDX-1/bzr1-1D*, *SRDX-9/bzr1-1D*, *SRDX-11/bzr1-1D*, *SRDX-4/bzr1-1D*) to explore whether their thermotolerance was significantly different from their own background after heat stress treatment. The expression level of *ERF49-SRDX* in these transgenic lines had basically the same fold change relative to its own background (Col or *bzr1-1D*), that is, the degree of inhibition of *ERF49* expression had the same level in the transgenic lines as the background (Fig. 6a). The results of survival rate analysis after heat stress treatment showed that *ERF49*-*SRDX*/*bzr1-1D* was strongly increased compared with *ERF49*-*SRDX*/Col. However, the trend of survival rate change of *ERF49*-*SRDX*/Col relative to Col-0 was the same as that of *ERF49*-*SRDX*/*bzr1-1D* relative to *bzr1-1D* (Fig. 6b, c). Furthermore, we also examined the thermotolerance of *ERF49* transgenic plants in the presence of 2, 4-eBL or BRZ (brassinazole, BR biosynthesis inhibitor). The statistical results showed that in the presence of 2, 4-eBL, the survival rate of plants generally increased after high temperature treatment. Exogenous application of 2, 4-eBL resulted in higher survival rate of *ERF49-SRDX* plants, while the survival rate of *ERF49-OX* plants was not significant different from wild-type (Additional file 1: Fig. S6a). Conversely, in the presence of BRZ, the survival rate of plants decreased at high temperature in general (Additional file 1: Fig. S6b). Compared with wild-type, the survival rate of overexpressed transgenic plants significantly decreased, while that of *ERF49-SRDX* transgenic plants significantly increased (Additional file 1: Fig. S6b). Taken together, these results suggest that *ERF49* is involved in the responsive to high temperature through the BR pathway.

### The HSPs are in downstream of BR-mediated ERF49 regulated heat stress response

**Fig. 7:**
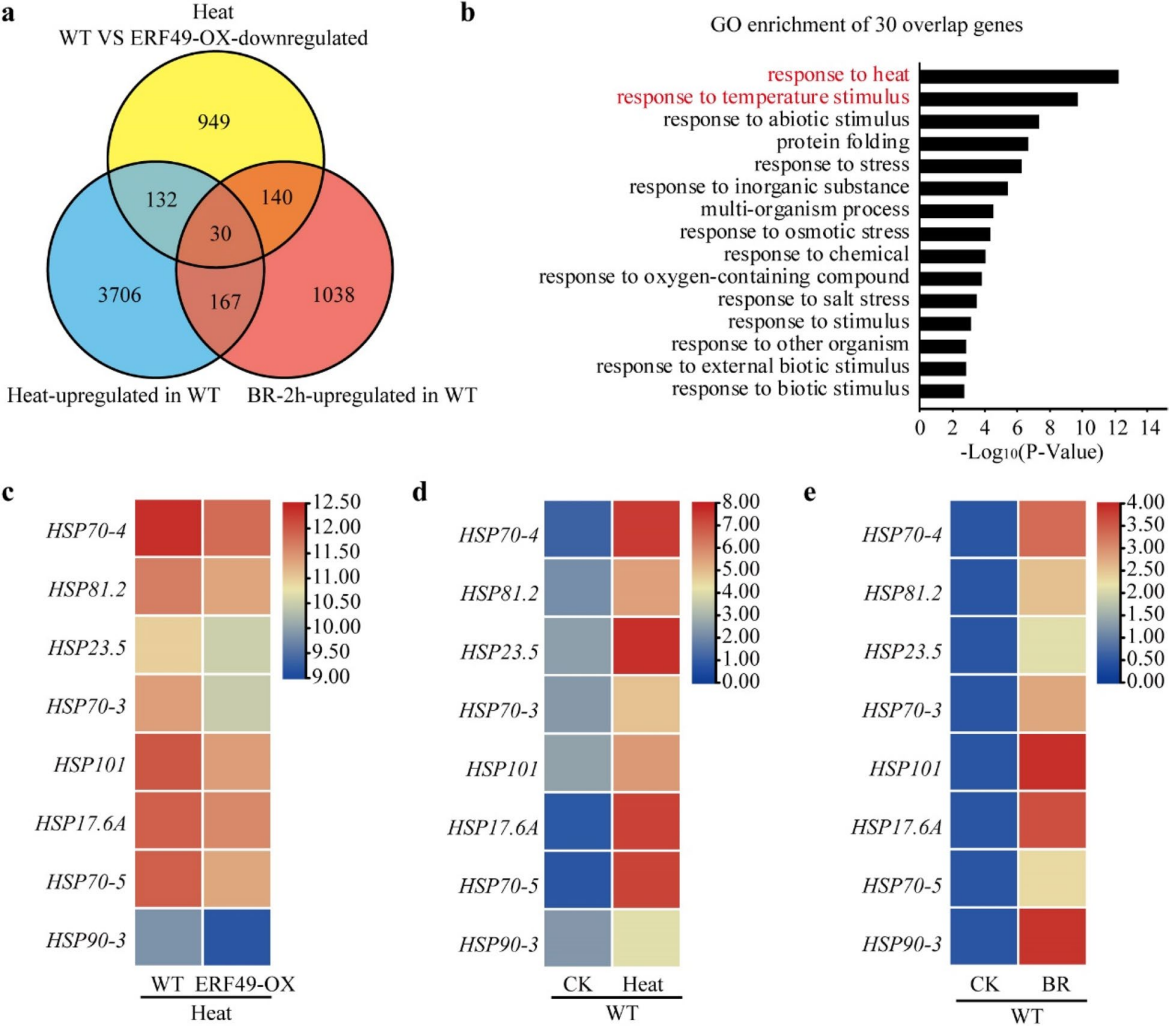
The *HSPs* are in downstream of BR and ERF49 in regulating heat stress response. **a** Venn diagram showing the overlap genes downregulated in *ERF49*-overexpression lines under heat stress and upregulated in wild-type after heat and BR treatment. **b** GO analysis of 30 overlap genes downregulated in *ERF49*-overexpression lines under heat stress and upregulated in wild-type after heat and BR treatment. **c–e** Heatmap of *HSP* genes in response to heat or temperature stimulus in WT and *ERF49*-overexpression plants (**c**), heat treatment (**d**), and BR treatment (**e**), respectively

We next performed RNA-seq analysis of *ERF49*-overexpression lines after heat stress treatment. The RNA-seq of *ERF49*-overexpression lines was analyzed with published transcriptome data of heat and BR treatment in wild-type to further investigate the downstream BR-mediated ERF49 in regulating heat stress response [40, 41]. There are 1251 genes downregulated in ERF49 overexpression lines after heat treatment (Additional file 2). The 4125 and 1375 genes were upregulated in wild-type after heat and BR treatment, respectively (Additional file 3 and Additional file 4). There are 30 overlap genes downregulated in *ERF49*-overexpression lines under heat treatment and upregulated in wild-type after heat and BR treatment (Fig. 7a and Additional file 5). Gene Ontology (GO) enrichment analysis of 30 overlap genes showed that the heat and temperature response terms are the most enriched terms (Fig. 7b). There are eight *HSP* genes in these two enriched terms and all of them are downregulated in *ERF49* overexpression lines under heat treatment (Fig. 7c) and upregulated in wild-type after heat and BR treatment (Fig. 7d, e). These results further confirmed that BR-mediated ERF49 regulates heat stress response by negatively regulating expression of *HSP* genes.

**Fig. 8:**
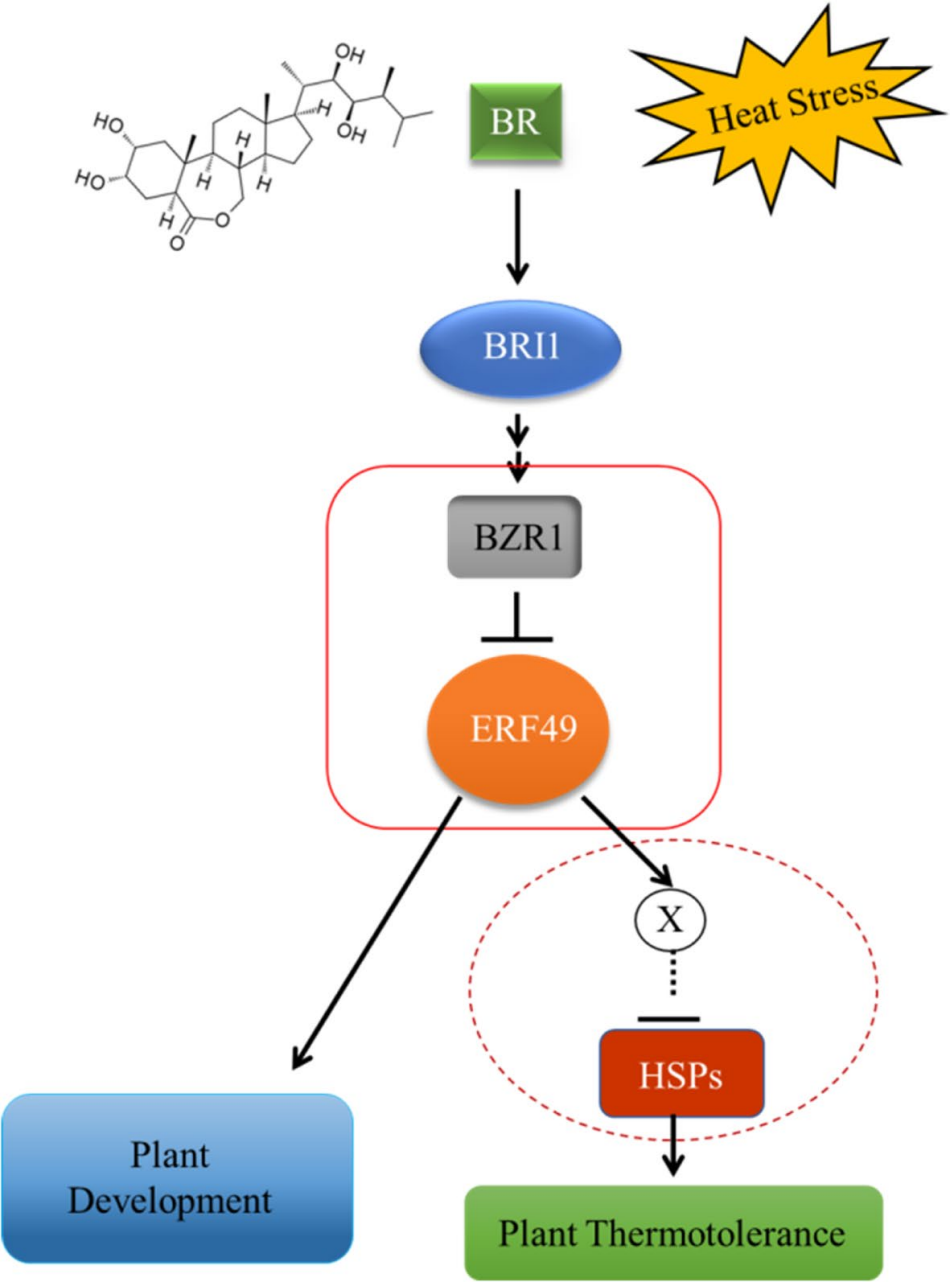
A model for ERF49-mediated BR regulation of heat stress tolerance. As a transcriptional activator, ERF49 positively regulates the downstream targets of BR signal transduction to promote plant development. When plants exposed to heat stress, the expression of *ERF49*, a direct target gene of BZR1, was inhibited. Meanwhile, ERF49 may activate of downstream HSPs-associated suppressors (X) in BR signal transduction, which directly or indirectly inhibit the expression of *HSPs* under high temperature stress

Based on the data collected above, we propose a working model of BR-mediated heat stress response mechanism in which BR promotes plant thermotolerance through BZR1 directly inhibiting the expression of *ERF49*. In addition, *ERF49* indirectly represses the *HSPs* expression to regulate the heat stress tolerance pathway (Fig. 8).

## Discussion

BR, an important plant hormone, is essential in plant responses to stress, such as high temperature, drought, salinity, and cold [15, 16]. One study reported that BR increases the basal heat resistance of *A. thaliana* seedlings [28], but the molecular mechanism of how BR mediates plant response to heat stress is still not clear. By analyzing thermotolerance of Col-0 treated with 2, 4-eBL and the response of BR-related mutants to high temperature, we found that BR enhances the tolerance of plants to high temperature in *A. thaliana* (Fig. 1).

The BES1 (BRI1-EMS-SUPPRESSOR 1) and BZR1 (BRASSINAZOLE RESISTANT 1) are two key transcription factors in BR signaling pathway which directly bind to thousands of BR-responsive gene promoters after dephosphorylation and control their transcription. When BRs are absent, the GSK3-like kinase BIN2 (BRASSINOSTEROID INSENSITIVE 2) phosphorylates BES1 and BZR1 proteins and inactivates these two factors [16]. Recent studies reported that the addition of BR increased the thermotolerance of *Arabidopsis*, and BRASSINOSTEROID INSENSITIVE 2 (BIN2) plays a critical role in the regulation of plant heat stress responses [42]. The BES1, as a transcription regulator, can be dephosphorylated and directly bind to heat shock element of heat stress transcription factors to regulate the expression of *HSFs* to respond to heat shock [43]. The BZR1 regulates growth and development of plants by regulating expression of downstream target genes [20, 21]. Several studies have found that BZR1 and PIF4 form a heterodimer to jointly regulate the expression of downstream target genes and participate in the response to high temperature [22]. Our study also revealed that BR contributes to thermotolerance of *Arabidopsis* and identified a new direct target gene of BZR1, *ERF49*, which regulates heat stress response via indirectly regulating the expression of heat stress-related proteins.

ERF49, also named DREB2D according to the phylogenetic relationship, is a member of the DREB2 subfamily of AP2/ERF domain transcription factors [23]. *Arabidopsis* DREB2 transcription factors have been reported to be involved in various abiotic stress processes [44, 45]. The DREB2A was rapidly and transiently induced under heat stress treatment at 37 °C, resulting in the expression of many transcription factors or molecular chaperones encoded by heat shock response genes [46]. And the expression of *HSFA3* was directly regulated by DREB2A under heat stress [46, 47]. Others previously showed that DREB2C is involved in heat responses and functions as a transcriptional activator of *HSFA3* during the heat stress response [48, 49]. DREB2C has also been reported to play a regulatory role in ABA and stress response [50]. However, the functions of DREB2 family transcription factors except DREB2A and DREB2C are still unclear. Here, our results suggested that DREB2D (ERF49) is also involved in heat response. We showed that *ERF49-SRDX* lines exhibit the phenotype of heat stress tolerance (Fig. 3), indicating that ERF49 plays a negative regulatory role in plant thermotolerance. Furthermore, overexpression of *ERF49* reduced the induction of *HSFA2* and *DREB2A* by heat stress, indicating that ERF49 may play a regulatory role in heat stress by regulating the expression of HSFA2. The *HSFA2* is a heat-inducible gene, which was reported to drive the transcriptional memory after heat stress via forming heteromeric complexes with HSFA3 and additional HSFs [51]. Recent research also reported that the HSFA2 directly activates the H3K27me3 demethylase REF6, which in turn de-represses HSFA2 and transmit plant thermotolerance [52]. Further investigation is needed into the specific regulatory mechanisms between ERF49 and HSFA2.

ERF49 functions as a positive regulator in the BR signal transduction pathway, because the dominant inhibitory mutant of EFR49 suppresses the phenotype of *bzr1-1D* (Fig. 5a). Why is the positive regulator ERF49 repressed by BRs and BZR1 in *bzr1-1D*? One possibility is that it represents a feedback regulatory mechanism to maintain the balanced level of BRs or BR signaling at which BZR1 regulates plant development through ERF49. This result is similar to the role of myeloblastosis family transcription factor-like 2 (MYBL2) in the BR signal transduction pathway [53]. The ERFs are key regulators in various biotic or abiotic stress response and capable of binding to different cis-element for different stress response, and for pathogen resistance response, the ERFs were reported to bind to GCC box of downstream genes [54]. For salt, drought, and heat stress response, the ERFs activate target genes for stress response by binding to a specific *cis*-acting dehydration-responsive element (DRE) (CCGAC) [25]. Our results suggested that ERF49 participates in the plant response to high temperature mainly through the BZR1 pathway (Fig. 6). The downstream target of ERF49 and the regulatory mechanism of ERF49 and its target for heat stress response need to be further investigated in the future.

As the direct target gene of BZR1, the expression of ERF49 positively regulates the activity of the downstream targets after being repressed by BZR1 (Fig. 5d). However, we found that ERF49 negatively regulates the activity of *HSPs* under high temperature (Additional file 1: Fig. 2). Why the transcriptional activator ERF49 represses the expression of *HSPs*, and thus negatively regulates the response to heat stress in *A. thaliana*? We speculate that the repression of *HSPs* by ERF49 is not a direct effect. One possibility is that ERF49 indirectly represses the *HSPs* expression by positively regulating the downstream HSPs-associated suppressors of BR signaling transduction (Fig. 8). Taking all of our data together, we hypothesized that BZR1 repressed the activity of downstream HSPs-associated suppressors by directly repressing the expression of ERF49, thereby indirectly promoting the expression of *HSPs* under high temperature stress, thus conferring the heat stress resistance in *A. thaliana* (Fig. 8).

## Conclusions

Our results provide an updated model for the thermotolerance of plants in *A. thaliana*. It is of great significance to further study the downstream target genes of ERF49 under high temperature stress. Moreover, other unknown pathways may also be involved in the response process of ERF49 to high temperature. Accordingly, it will be interesting to further explore the exact mechanism responsible for plant thermotolerance.

## Methods

### Plant materials and growth conditions

*Arabidopsis thaliana* ecotype Columbia (Col-0) was used in this study as a wild-type control. *bzr1-1D* and *BZR1pro::BZR1-GFP* are in the Col-0 ecotype background and both were described previously [19]. *bri1-5* is in the Wassilewskijia (WS) ecotype background [55]. *Arabidopsis* seeds were sterilized with 75% ethanol plus 0.01 % Triton X-100 for 15 min, then rinsed with 95% ethanol, and dried in a hood. Sterilized seeds were then sown on half-strength Murashige and Skoog (1/2 MS) medium, and plates with seeds were maintained at 4 °C for 2 days to break dormancy prior to transfer to a culture room at 22 °C under dark/light cycles of 8/16 h for 1 week. Positive transgenic seedlings, screened by applying hygromycin B in the medium, were transferred and grown in a controlled culture room with the same dark/light cycle conditions. For plant treatment, 2, 4-eBL stock solution was diluted to the working concentration in 1/2 MS medium.

### Hypocotyl assay and survival assay under heat stress

In this study, 2, 4-eBL was used in all BR treatments. To detect seedling response to BR (2, 4-eBL), seeds were spotted on 1/2 MS medium containing 10 nM, 100 nM, 1 μM, and 10 μM 2, 4-eBL, vernalized for 2 days, and vertically cultured on 1/2 MS medium for 7 days. Phenotype analysis of hypocotyls was described previously [19].

For the survival assay under heat stress, 7-day-old seedlings grown on 1/2 MS medium were transferred to 42 °C for 3 h in dark, and then transferred into the culture room at 22 °C under dark/light cycles of 8/16 h for 5 days. In the statistics of survival rate, we counted the number of fully etiolated seedlings, partially etiolated seedlings, and non-etiolated seedlings after high temperature treatment. Survival rate = (number of non-etiolated seedlings + 1/2 number of partially etiolated seedlings) / total number of seedlings.

### Plasmid construction and generation of transgenic plants

To obtain the overexpression vector of *ERF49 (ERF49-OX)*, a 621-bp genomic fragment containing full-length *ERF49* open reading frame (ORF) was amplified by PCR and ligated into pENTRTM/SD/D-TOPO vector, and then ligated into pMDC32 by recombinant cloning [56]. To get the dominant repressor of *ERF49 (ERF49-SRDX)*, the full-length cDNA sequence was amplified by PCR. Then the segment was ligated into p35S-SRDX vector between *BamH*I and *Spe*I sites. To get localized expression of *ERF49*, a 2582-bp (without terminator codon) genomic segment containing promoter and gene sequence was amplified by PCR and ligated into pENTRTM/SD/D-TOPO vector, and then ligated into pMDC163 to produce *ERF49pro::gERF49-GUS* [56]. To obtain the vector of *35S::ERF49-GFP*, a 621-bp genomic fragment containing full-length *ERF49* open reading frame (ORF) was amplified by PCR and ligated into pENTRTM/SD/D-TOPO vector, and then ligated into pMDC83 by recombinant cloning [56]. The constructs were transformed into the *Agrobacterium tumefaciens* strain GV3101 and then introduced into *A. thaliana* plants via floral dip method [57].

The *ERF49* promoter fragment with mutant BRRE motif (CGTCTC to TTTTTT) was synthesized via a biological company (ShengGong, Shanghai, China). For the dual-luciferase reporter assay, a 1.6-kb fragment upstream of the *ERF49* translational start code and the corresponding DNA fragments with intact or mutated BRRE *cis*-element variant were PCR amplified and inserted into the *Sal*I*-BamH*I site of the pGreenII0800-LUC vector, generating *ERF49pro::LUC*. The *BZR1* open reading frame was PCR amplified and inserted into the *Mfe*I-*Kpn*I site of pUC18-3HA to produce *35S::BZR1* for the analysis in *Arabidopsis* protoplasts and inserted into the pGreenII 62-SK vector for the analysis in tobacco to produce *pGreenII 62-SK::BZR1*.

### Total RNA extraction and quantitative RT-qPCR analysis

Total RNA was extracted from *Arabidopsis* seedlings using Trizol reagent (Invitrogen, Carlsbad, California, USA). First-strand cDNA was synthesized with a reverse transcript kit (Vazyme, China) and used as RT-qPCR templates. Quantitative real-time PCR analyses were carried out on an ABI7500 (Applied Biosystems, Foster City, California, USA) using the SYBR@ Green reagent (Toyobo, Osaka, Japan) according to the manufacturer’s instructions. The RT-qPCR was repeated at least three times using samples harvested separately. The *UBC30* gene was used as an internal reference. The primer sequences used for RT-qPCR are in Additional file 1: Table S1.

### Assays of GUS activity

Histochemical GUS assays were performed in a staining solution containing 0.5 mg mL^−1^ 5-bromo-4-chloro-3-indolyl glucuronide (X-Gluc) in 0.1 M Na_2_HPO_4_, pH 7.0, 10 mM Na_2_EDTA, 0.5 mM potassium ferricyanide/ferrocyanide, and 0.06 % Triton X-100 [58]. Samples were infiltrated under vacuum for 10 min and then incubated at 37 °C. After overnight incubation, the staining buffer was removed, and the samples were cleared in 70 % ethanol. All observations by light microscopy were made with the Olympus BX51 microscope system.

### Chromatin immunoprecipitation-qPCR

Chromatin immunoprecipitation (ChIP)-qPCR experiments were performed following the protocol described previously [36], using 2-week-old light-grown wild-type and *BZR1pro::BZR1-CFP* (*w2c*) transgenic *Arabidopsis* seedlings. An affinity-purified anti-GFP polyclonal antibody was used to immunoprecipitate the BZR1 protein-DNA complex, and the precipitated DNA was analyzed by RT-qPCR using the SYBR@ Green reagent (Toyobo, Osaka, Japan). Results were presented as the ratio of the amount of DNA immunoprecipitated from *BZR1-CFP* samples to that of the control samples (wild-type). The *UBC30* and *PP2A* genes were used as negative controls. The ChIP-qPCR experiments were performed three times, from which the means and standard deviations were calculated. The primer sequences for ChIP-qPCR are in Additional file 1: Table S1.

### Protoplast transient expression assay

For transient expression of *ERF49* by BZR1, the reporter plasmid *ERF49pro::LUC* and *35S::BZR1* effector or vector control were co-transformed into *Arabidopsis* protoplasts. *Arabidopsis* protoplasts were isolated as described previously [59]. The *ERF49pro::LUC* reporter gene construct contains the native sequence of the *ERF49* promoter fused to luciferase (LUC). Luminescence activities of firefly and *Renilla* were measured using Dual-Luciferase Reporter Assay System reagent in a Modulus Luminometer/Fluorometer equipped with a luminescence kit (Promega). The relative reporter expression level was expressed as the LUC firefly/LUC *Renilla* ratio [60, 61].

### Dual-luciferase reporter assay in tobacco

The pGreenII 0800-LUC fusion constructs carrying the promoters or the corresponding fragments of *ERF49* and the *pGreenII 62-SK::BZR1* construct were individually introduced into *Agrobacterium tumefaciens* strain GV3101. The *Agrobacterium* cells containing the 35S::BZR1 and the LUC fusion constructs were mixed at a ratio of 1:1 and infiltrated into *Nicotiana benthamiana* leaves [62]. After incubated for 3 days, the intensity of the firefly luciferase bioluminescence was measured using an imaging system (Xenogen IVIS 100, PerkinElmer). Luciferase activity was also detected using the Dual-Luciferase Reporter Assay System (Promega Corp., Fitchburg, WI, USA) according to the manufacturer’s protocol.

### Yeast one-hybrid assay

*BZR1* cDNA was inserted into the unique *EcoR*I and *PspX*I sites of the pJG45 vector. The *ERF49* promoter and the corresponding fragments with intact or mutated BRRE *cis*-element variant were amplified from *Arabidopsis* and subcloned into the pLacZi vector [63] to drive lacZi reporter gene expression. The constructs were transformed into yeast strain EGY48. Positive transformants grown on the SD/-Trp/-Ura medium (Clontech, Mountain View, CA, USA) were transferred to the selection medium containing raffinose, galactose, and 5-Bromo-4-chloro-3-indolyl-b-D-galactopyranoside (Amresco, Solon, OH, USA) for blue color development. β-galactosidase activity was assayed by hydrolysis of ortho-nitrophenyl-β-D-galactopyranoside (ONPG), as well as measuring absorbance for the released ortho-nitrophenyl (ONP) compound on a spectrophotometer at 415 nm. Relevant PCR primer sequences are given in Additional file 1: Table S1.

### Subcellular localization analysis

To determine the subcellular localization of gene products, the fusion construct (*35S::ERF49-GFP*) was transformed into tobacco leaf cells. Green fluorescence was observed under a confocal microscope (Leica TCS SP5, Wetzlar, Germany).

### Expression analysis

The expression analysis of *BZR1* and *ERF49* was performed by the Arabidopsis eFP Browser (http://bar.utoronto.ca/efp/cgi-bin/efpWeb.cgi). The comparable developmental map was generated by typing appropriate parameters as described in original eFP Browser paper [64].

### RNA-seq analysis

The *ERF49* overexpression and Col plants were grown into 42 °C for 3 h, and about 100 mg leaves were used to extract total RNA. The 3 μg RNA was used to construct RNA-seq libraries and RNA-seq was repeated three times. Differentially expressed genes (DEGs) were identified with Fold Change ≥ 1.5 and adjusted *p*-values *<* 0.05. In addition, the published heat and BR treatment RNA-seq data were obtained [40, 41]. The GO analysis of genes downregulated in *ERF49* overexpression lines and upregulated in BR and heat treatment plants were performed using agriGO v2.0 [65].

## Supplementary Information

**Additional file 1: Figure S1.** Expression analysis of *BZR1* and *ERF49* in different tissues. **Figure S2.** Relative expression level of *HSPs*, *HSFs* and *DREB2A* in *ERF49* transgenic lines. **Figure S3.** Bioinformatics analysis for binding of BZR1 to the *ERF49* promoter. **Figure S4.** The analysis of hypocotyl elongation in the *ERF49* transgenic plants with 2, 4-eBL treatment. **Figure S5.** Dominant-negative *ERF49* increases thermotolerance in *bzr1-1D*. **Figure S6.** Thermotolerance analysis of *ERF49* transgenic lines in the presence of 2, 4-eBL and BRZ. **Table S1.** Primers used in this study.

**Additional file 2.** Detailed information of genes downregulated in ERF49 overexpression lines after heat treatment.

**Additional file 3.** Detailed information of genes upregulated after heat treatment.

**Additional file 4.** Detailed information of genes upregulated after BR treatment.

**Additional file 5.** Detailed information of 30 overlap genes downregulated in ERF49 overexpression lines and upregulated after heat and BR treatment.

**Additional file 6.** Combined raw data, with each figure on a spreadsheet.

## Data Availability

All data generated or analyzed during this study are included in this published article, its supplementary information files and publicly available repositories. RNA-seq datasets are available from the NCBI sequence Read Archive BioProject (PRJNA891032).

## Abbreviations

ERF: Ethylene responsive factor
BZR1: Brassinazole resistant 1
BR: Brassinosteroid
HSF: Heat shock factor
HSP: Heat-shock protein
bzr1-1D: Brassinazole-resistant 1-1D
ROS: Reactive oxygen species
HSE: Heat shock element
PIF4: Phytochrome interacting factor 4
eBL: Epi-brassinosteroid
BRBT: BR-regulated BZR1 target
NLS: Nuclear localization sequence
BES1: BRI1-EMS-Suppressor 1
BIN2: Brassinosteroid insensitive 2
MYBL2: Myeloblastosis family transcription factor-like 2
DRE: Dehydration-responsive element
BRZ: Brassinazole
WS: Wassilewskija
ORF: Open reading frame
LUC: Luciferase
ChIP-chip: Chromatin immunoprecipitation microarray
OPNG: Ortho-nitrophenyl-β-D-galactopyranoside
ONP: Ortho-nitrophenyl
GO: Gene Ontology
EV: Empty vector
