# Supplementary material for: ERF49 mediates brassinosteroid regulation of heat stress tolerance in Arabidopsis thaliana
Source: BMC Biol. 2022 Nov 10;20:254. doi: 10.1186/s12915-022-01455-4 (PMC9650836; doi:10.1186/s12915-022-01455-4)
Supplement: Supplementary file 1 — Additional file 1: Figure S1. Expression analysis of BZR1 and ERF49 in different tissues. Figure S2. Relative expression level of HSPs, HSFs and DREB2A in ERF49 transgenic lines. Figure S3. Bioinformatics analysis for binding of BZR1 to the ERF49 promoter. Figure S4. The analysis of hypocotyl elongation in the ERF49 transgenic plants with 2, 4-eBL treatment. Figure S5. Dominant-negative ERF49 increases thermotolerance in bzr1-1D. Figure S6. Thermotolerance analysis of ERF49 transgenic lines in the presence of 2, 4-eBL and BRZ. Table S1. Primers used in this study. [file 12915_2022_1455_MOESM1_ESM.docx]

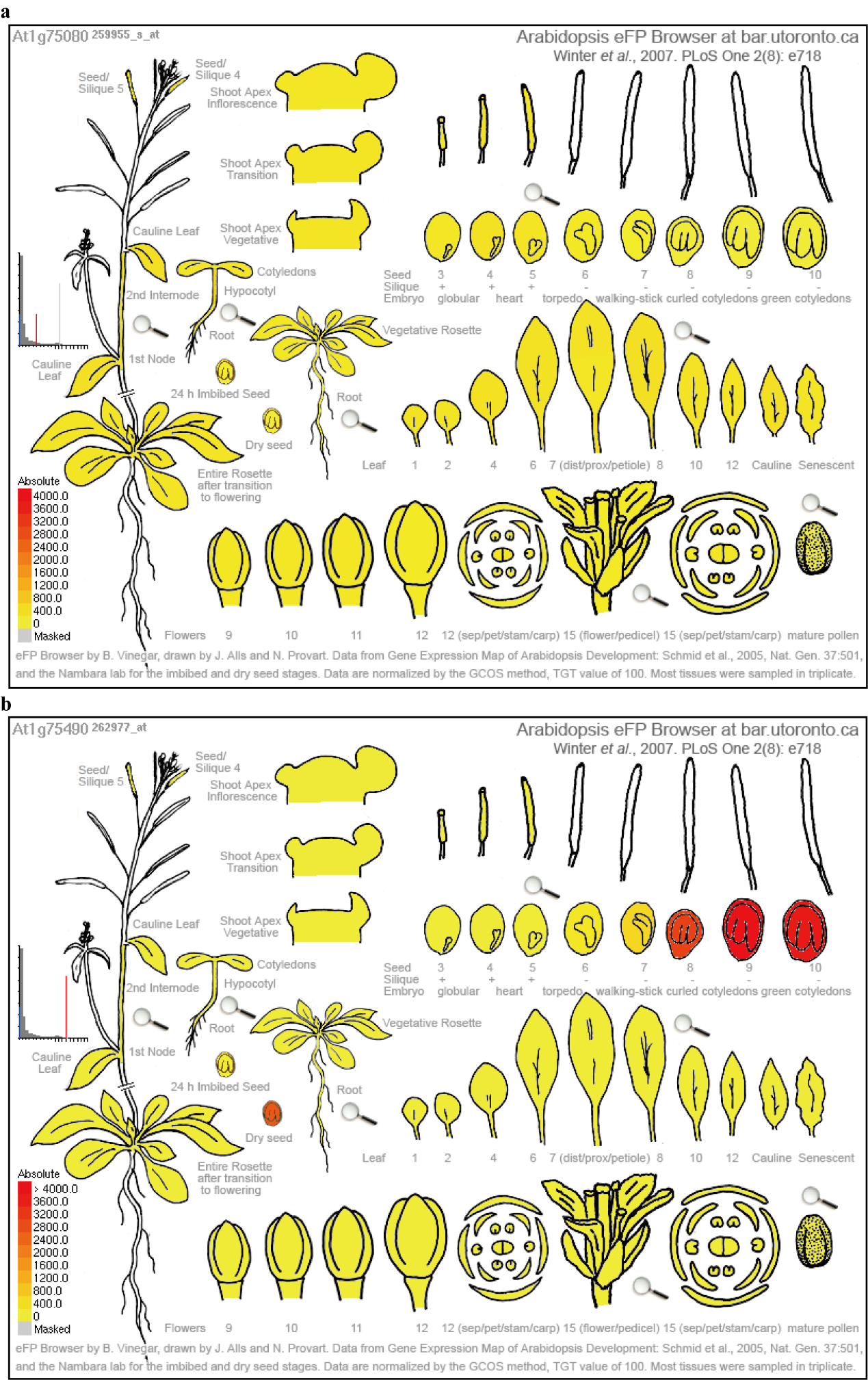


**Figure. S1 Expression analysis of *BZR1* and *ERF49* in different tissues**

Absolute expression of *BZR1* (At1g75080) (**a**) and *ERF49* (At1g75490) (**b**) in the Arabidopsis developmental map from Arabidopsis eFP Browser (http://bar.utoronto.ca/)^[65]^.


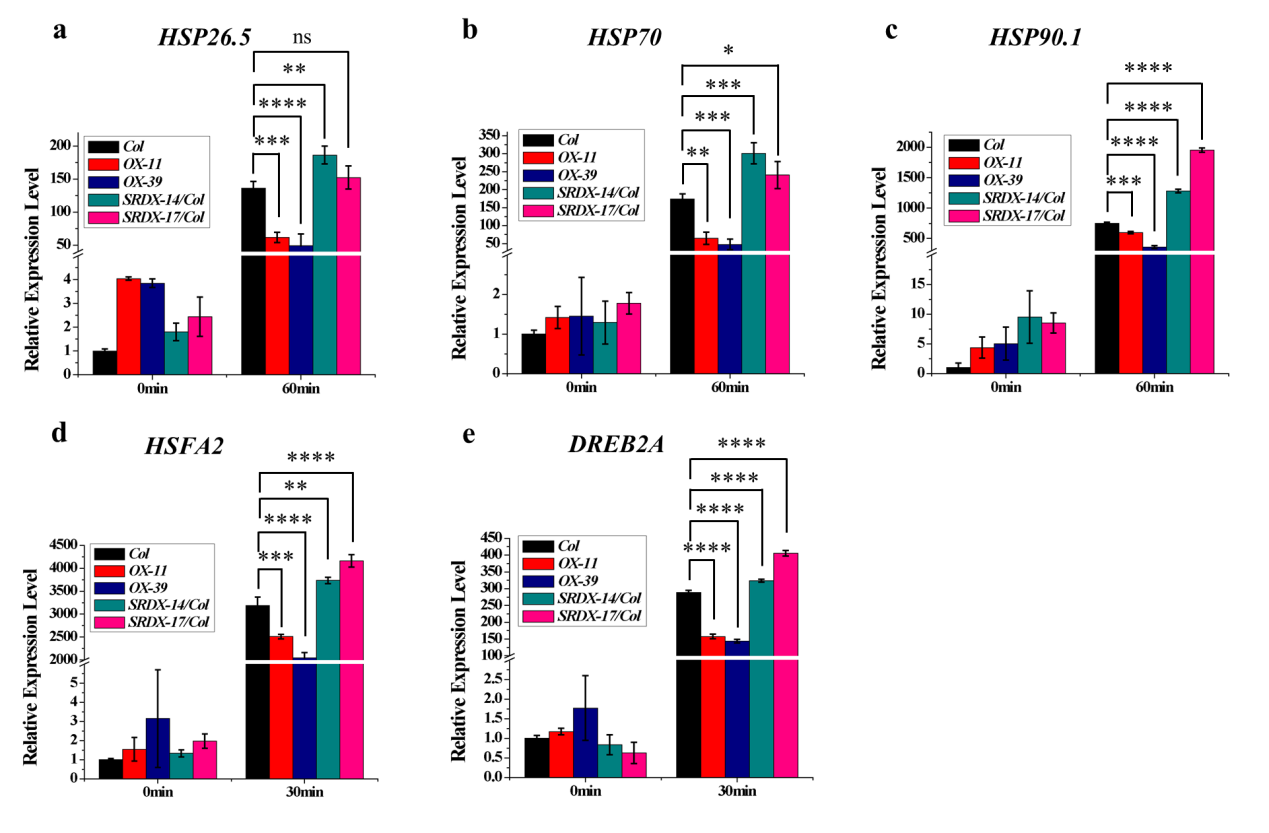


**Figure. S2 Relative expression level of *HSPs*, *HSFs* and *DREB2A* in *ERF49* transgenic lines**

Relative expression levels of *HSP26.5* (**a**), *HSP70* (**b**), *HSP90.1* (**c**), *HSFA2* (**d**), and *DREB2A* (**e**) in *ERF49* transgenic lines after heat stress treatment for 0 min and 60 min. Asterisks represent statistical significance (ns, no significant, P > 0.05; *, P < 0.05; **, P < 0.01; ***, P < 0.001; ****, P < 0.0001; one-way ANOVA followed by post hoc Tukey’s multiple comparison test). *UBC30* was used as a reference gene. Numerical data is provided in Additional file 6. The experiments were repeated three times, error bars indicate standard deviation.


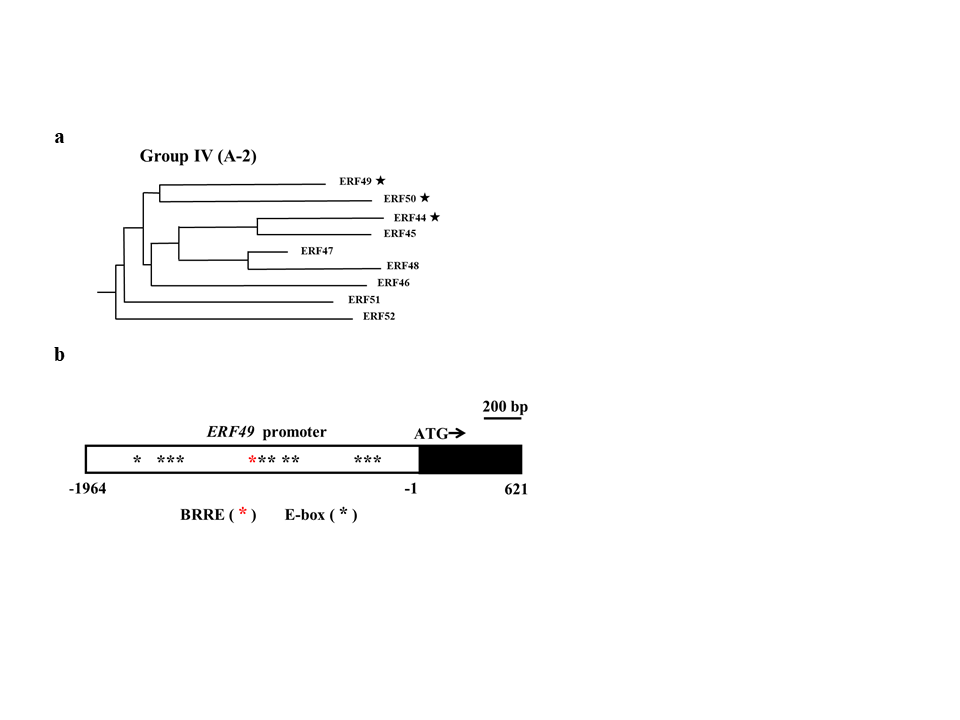


**Figure. S3 Bioinformatics analysis for binding of BZR1 to the *ERF49* promoter**

**a**. Analysis of BZR1’s direct target genes in group IV of the ERF family. Three of the nine genes in this group are direct targets of BZR1 and are marked with stars. **b**. Diagram depicting the putative promoter (open box) and the exon (black box) of the *ERF49* gene. Black and red asterisks indicate the positions of putative E-box and BRRE motifs, respectively.


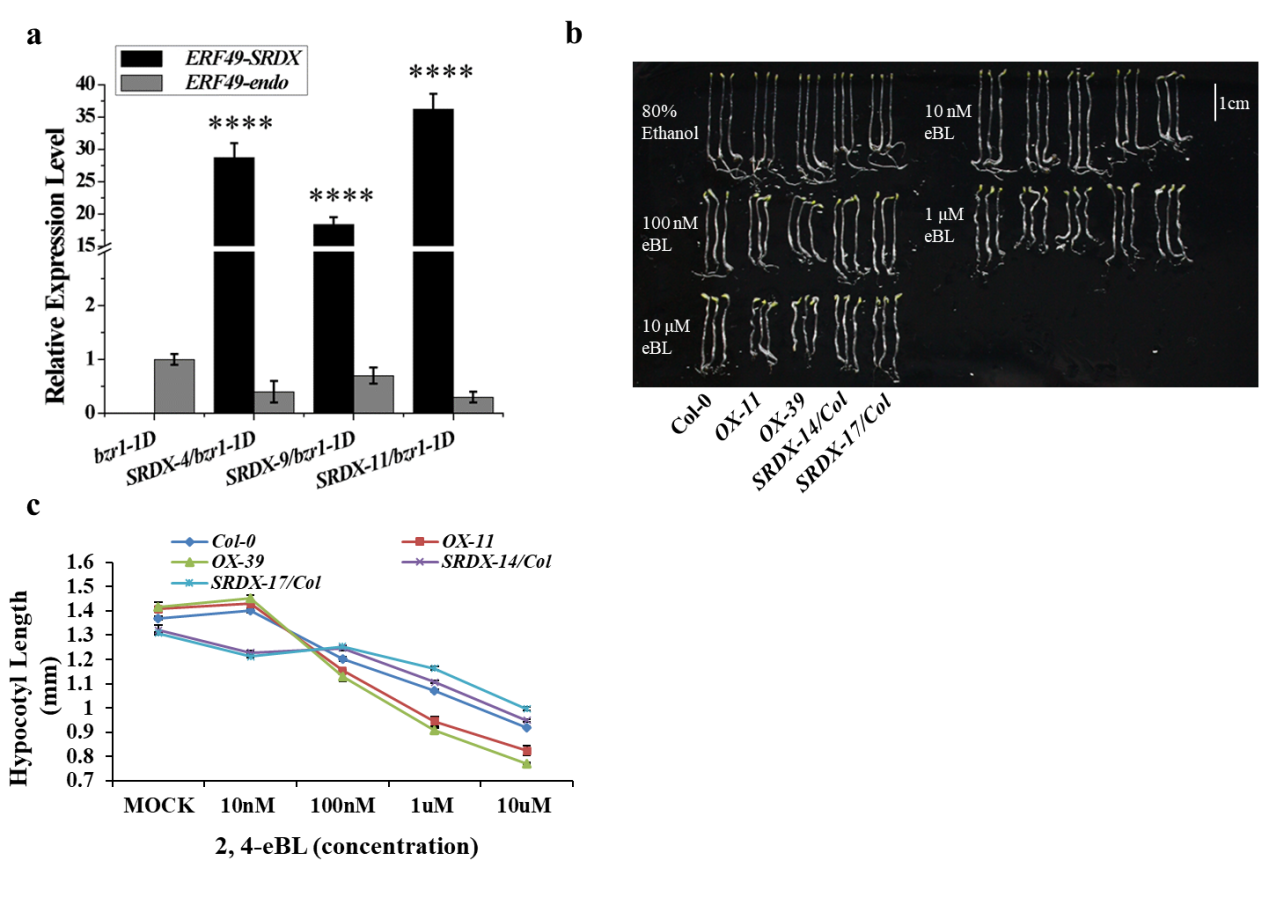


**Figure. S4 The analysis of hypocotyl elongation in the *ERF49* transgenic plants with 2, 4-eBL treatment**

**a**. The relative expression level of ERF49 in three ERF49 dominant-negative transgenic lines under bzr1-1D background (SRDX-4/bzr1-1D, SRDX-9/bzr1-1D, and SRDX-11/bzr1-1D). Asterisks represent statistical significance (****, P < 0.0001; one-way ANOVA followed by post hoc Tukey’s multiple comparison test). UBC30 was used as a reference gene. Numerical data is provided in Additional file 6. The experiments were repeated three times, error bars indicate standard deviation. **b**. The hypocotyl elongation in the ERF49 transgenic plants with 2, 4-eBL or 80 % ethanol (as control) treatment. Seedlings were grown on 1/2 MS medium containing 80% ethanol and 10 nM, 100 nM, 1 μM, or 10 μM 2, 4-eBL in the dark for 7 days. Scale bar was 1 cm. **c**. Quantitative statistics of hypocotyl lengths from B, the hypocotyl lengths of over 30 seedlings were measured by Image J for each independent line, error bars represent standard deviation.


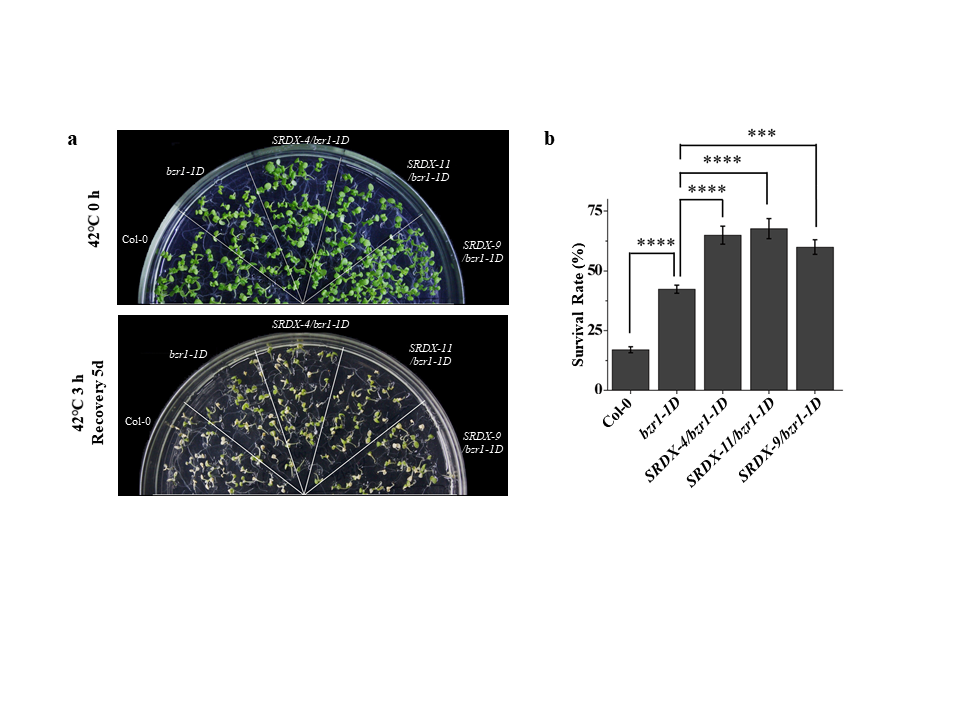


**Figure. S5 Dominant-negative *ERF49* increases thermotolerance in *bzr1-1D***

**a**. The phenotype of *ERF49* dominant-negative transgenic lines in *bzr1-1D* after heat stress treatment at 42 °C for 3 hours and recovery for 5 days. *bzr1-1D* was the control. **b**. The survival rates of *ERF49* dominant-negative transgenic lines in *bzr1-1D* after heat stress treatment at 42 °C for 3 hours and recovery for 5 days. 60-80 seedlings of each genotype from three biological replicates were counted. *bzr1-1D* was the control. Asterisks represent statistical significance (***, P < 0.001; ****, P < 0.0001; one-way ANOVA followed by post hoc Tukey’s multiple comparison test). Numerical data is provided in Additional file 6. The experiments were repeated three times, error bars indicate standard deviation.


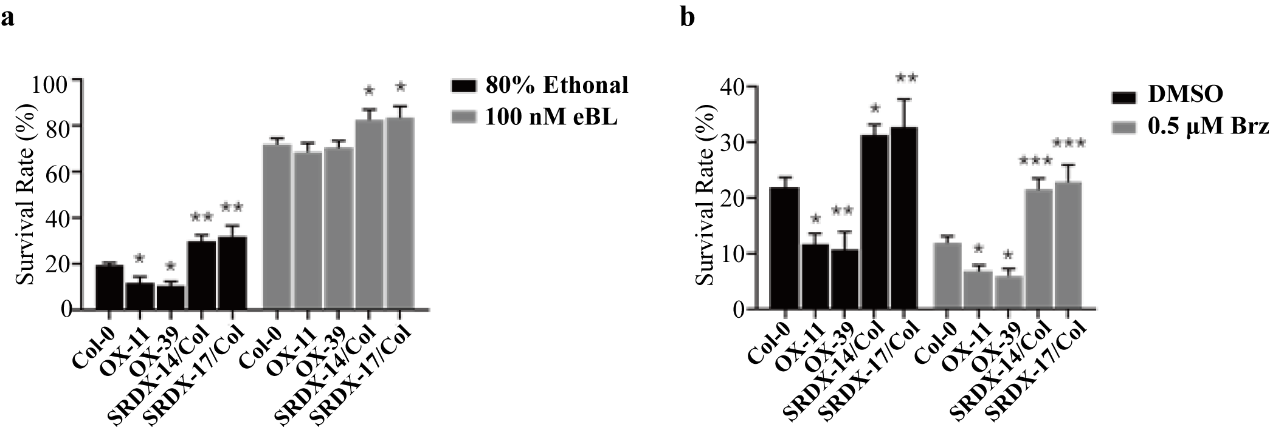


**Figure. S6 Thermotolerance analysis of *ERF49* transgenic lines in the presence of 2, 4-eBL and BRZ**

a. The survival rate of *ERF49* transgenic lines in presence of 100 nM 2, 4-eBL after heat stress treatment at 42°C for 3 hours and recovery for 5 days. b. The survival rate of *ERF49* transgenic lines in presence of 0.5 μM BRZ after heat stress treatment at 42°C for 3 hours and recovery for 5 days. 60-80 seedlings of each genotype were counted with three biological replicates. Error bars indicate standard deviation. Asterisks represent statistical significance (*, P < 0.05; **, P < 0.01; ***, P < 0.001; one-way ANOVA followed by post hoc Tukey’s multiple comparison test). Numerical data is provided in Additional file 6.

**Table. S1 Primers used in this study**

| Primer name | Sequence (5’- 3’) | Usage |
| --- | --- | --- |
| *ERF49*-SRDX-F | CGGGATCCGTCTTTGCCTTATCACTCACCA | Primers used for constructs |
| *ERF49*-SRDX-R | GGACTAGTAAGTGGGGAATGAAAGGAATC |  |
| *ERF49*-OX-F | CACCTAACCATTACCTCATGTC |  |
| *ERF49*-OX-R | AAGTGGGGAATGAAAGGAATC |  |
| *ERF49pro*-F | CACCTGGTAACCAATCTCAAAAGTCA |  |
| *ERF49pro*-R | CATGTACGGATGTTGATAACTCG |  |
| *ERF49pro*-LUC-F | ACGCGTCGACTGCAAAGACTGTCCTTAGTG |  |
| *ERF49pro*-LUC-R | CGCGGATCCCGAAAGTGATATTATGTGACG |  |
| *BZR1*-PUC-F | GAACCAATTGATGACTTCGGATGGAGCTACGTC |  |
| *BZR1*-PUC-R | CGGGGTACCTCAACCACGAGCCTTCCCAT |  |
| *ERF49pro*-LUC-F | CCGGAATTCCAAACTGAACTAAGTATCACGTC |  |
| *ERF49pro*-LUC-R | CGGGGTACCCGTTGTTTCTTATTAGCACC |  |
| *ERF49pro*-1-LUC-F | CCGGAATTCTGCAAAGACTGTCCTTAGTGG |  |
| *ERF49pro*-1-LUC-R | CGGGGTACCGTTATGATGGATTTGACTCCG |  |
| *ERF49pro*-2-LUC-F | CCGGAATTCAGATAAATTGGAACCGGAGC |  |
| *ERF49pro*-2-LUC-R | CGGGGTACCGTTCACTGAATCTTACTTCTCTGTC |  |
| *ERF49pro*-3-LUC-F | CCGGAATTCAGCCATAGCTTGATTTTGTT |  |
| *ERF49pro*-3-LUC-R | CGGGGTACCCGTTGTTTCTTATTAGCACC |  |
| *AtBZR1*-AD-F | CCGGAATTCATGACTTCGGATGGAGCTACGTC |  |
| *AtBZR1*-AD-R | CCGGCTCGAGCTCAACCACGAGCCTTCCCAT |  |
| *ERF49pro-F* (with BRRE motif)*:* | AGATAAATTGGAACCGGAGCTTTGTAGTTATCGGTTAAAAAGGCCGCCTTTTCACGTGTGTCTCGGCCACAAATGTTCTAAGACAGAGAAGTAAGATTCAGTGAAC |  |
| *ERF49pro-m* (with mutant BRRE motif)*:* | AGATAAATTGGAACCGGAGCTTTGTAGTTATCGGTTAAAAAGGCCGCCTTTTCATTTTTTTCTCGGCCACAAATGTTCTAAGACAGAGAAGTAAGATTCAGTGAAC |  |
| *AtBZR1*-JG45-F | GATTATGCCTCTCCCGAATTCATGACTTCGGATGGAGCTACG |  |
| *AtBZR1*-JG45-R | AGAAGTCCAAAGCTTCTCGAGACCACGAGCCTTCCCATTT |  |
| *ERF49pro-*LacZi-F | GGAATTCCACCTGGTAACCAATCTCAAAAGTCA |  |
| *ERF49pro-*LacZi-R | CCGCTCGAGCATGTACGGATGTTGATAACTCG |  |
| *ERF49pro-F/m-*LacZi-F | GGAATTCAGATAAATTGGAACCGGAGCTTT |  |
| *ERF49pro-F/m-*LacZi-R | CCGCTCGAGGTTCACTGAATCTTACTTCTCTGTCTTA |  |
| *AtBZR1*-62SK-F | CGCTCTAGAACTAGTGGATCCATGACTTCGGATGGAGCTACGT |  |
| *AtBZR1*-62SK-R | TCAGCGTACCGAATTGGTACCACCACGAGCCTTCCCATTTC |  |
| *ERF49pro*-0800-F | GGGGTACCCACCTGGTAACCAATCTCAAAAGTCA |  |
| *ERF49pro*-0800-R | CGGGATCCCATGTACGGATGTTGATAACTCG |  |
| *ERF49pro-F/m-*0800-F | GGGGTACCAGATAAATTGGAACCGGAGCTTTGT |  |
| *ERF49pro-F/m-*0800-R | CGGGATCCGTTCACTGAATCTTACTTCTCTGTCTTA |  |
| *ERF49-*qF  *ERF49-*qR  *ERF49-*SRDX*-*qF  *ERF49-*SRDX*-*qR  *ERF49-*endo*-*qF  *ERF49-*endo*-*qR  *UBC30-*qF  *UBC30-*qR  *SAUR_AC1-*qF  *SAUR_AC1-*qR  *CPD-*qF  *CPD-*qR  *XTR6-*qF  *XTR6-*qR  *RAB18-*qF  *RAB18-*qR  *RD29A-*qF  *RD29A*-qR | AGACTACACCAAGCAGCAACACC  TTGGATGAACACGGCGACTCAG  TGTCATCATGTGGAAGAGTGACA  CGAAACCCAAACGGAGTTCTAG  TCGCGGATGACCTTATCCATA  CGTGTCGAGAGGAGGAAGC  TCACTTCCCACCAGATTACCC  TCGACAGAAGAACCTTGGATACG  CGGTCTATGTAGGAGAGAATG  GTATCTGAGATGTGACTGTG  TTGCTCAACTCAAGGAAGAG  TGATGTTAGCCACTCGTAGC  TTACTCTATCCTCTGGAACC  GTAGGAAGCAGTGAAGGGAG  TCCAGCTCTAGCTCGGAGGATGA  GGATCCCATGCCGCCCATCG  GCCGGAATCTGACGGCGGTT  CCCGTCGGCACATCCTTGTCG | Primers used for Real-time PCR |
| UBC30-F  UBC30-R  PP2A-F  PP2A-R  DWF4-F  DWF4-R  CPD-F  CPD-R  ERF49-F  ERF49-R | CAAATCCAAAACCCTAGAAACCGA  AACGACGAAGATCAAGAACTGGGAA  AGCAGCACAACCCTCAACAG  CCAGATGTGCTAAAGACGGAG  GGGTTTGACTGTCCAGTTCGGTAAT  ACCCTTAGGATATGGGAAAAGGGTG  CCCCCCGTGTGCCCACTC  CCATTGAAGAAGAAGATGATGATGA  GAGCTTTGTAGTTATCGGTT  CACTGAATCTTACTTCTCTGTC | Primers used for ChIP-qPCR |
